# Supplementary figures and images for: Efficacy and cost of high-frequency IGRT in elderly stage III non-small-cell lung cancer patients
Source: PLoS One. 2021 May 27;16(5):e0252053. doi: 10.1371/journal.pone.0252053 (PMC8158910; doi:10.1371/journal.pone.0252053)

Distribution of IGRT Utilization By Patient

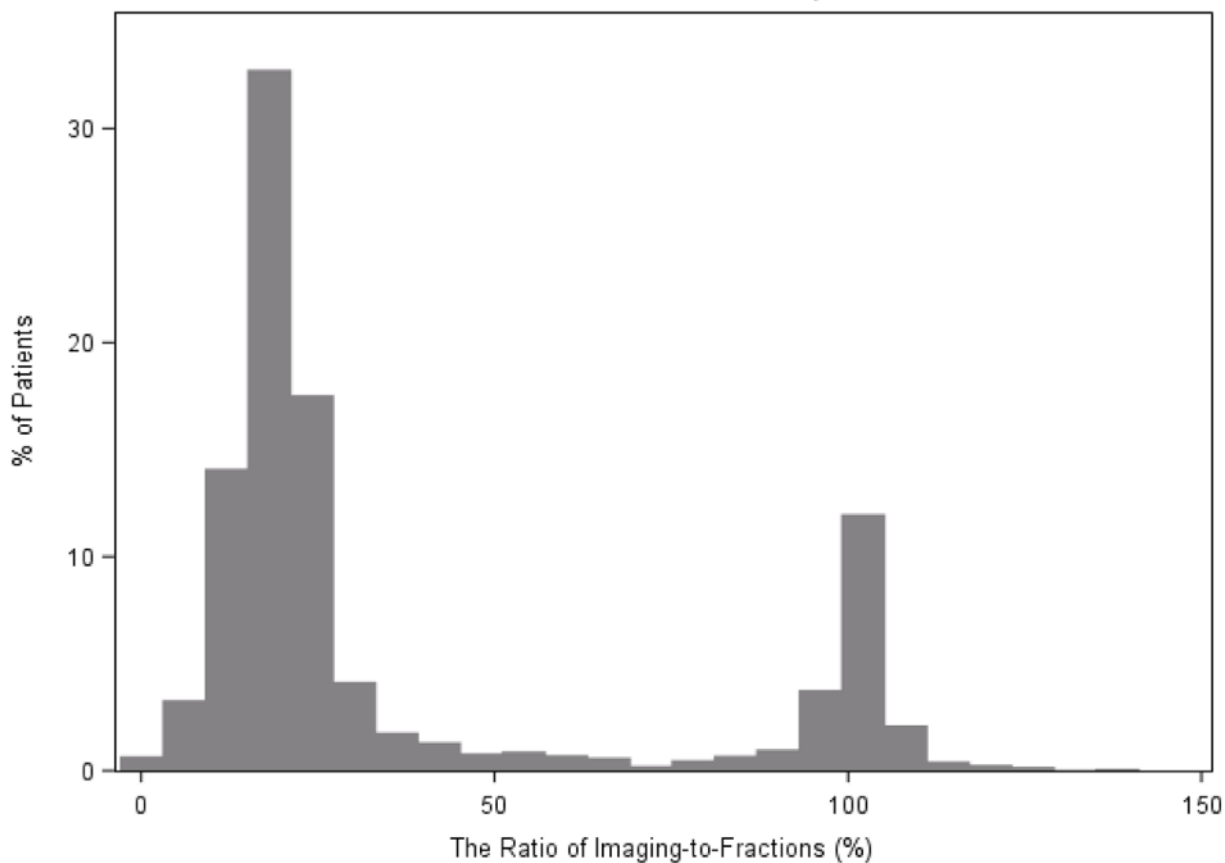

Supplement: S1 Fig — Histogram showing the % of patients using IGRT at different frequencies. X-axis represents the percentage of radiation fractions accompanied by image-guidance. Y-axis is the percent of patients in our cohort who received IGRT at that frequency. The distribution is bimodal, with most patient receiving image guidance with ~20% of radiation fractions or ~100% of radiation fractions. Some patients appear to receive image guidance with greater than 100% of radiation fractions because initial planning CT scans are often billed using the same code as IGRT and cannot be distinguished using billing, even though these CT scans are not accompanied by a radiation fraction. (PDF) [file pone.0252053.s001.pdf]

Popularity of Port/KV vs. CBCT for IGRT by Year

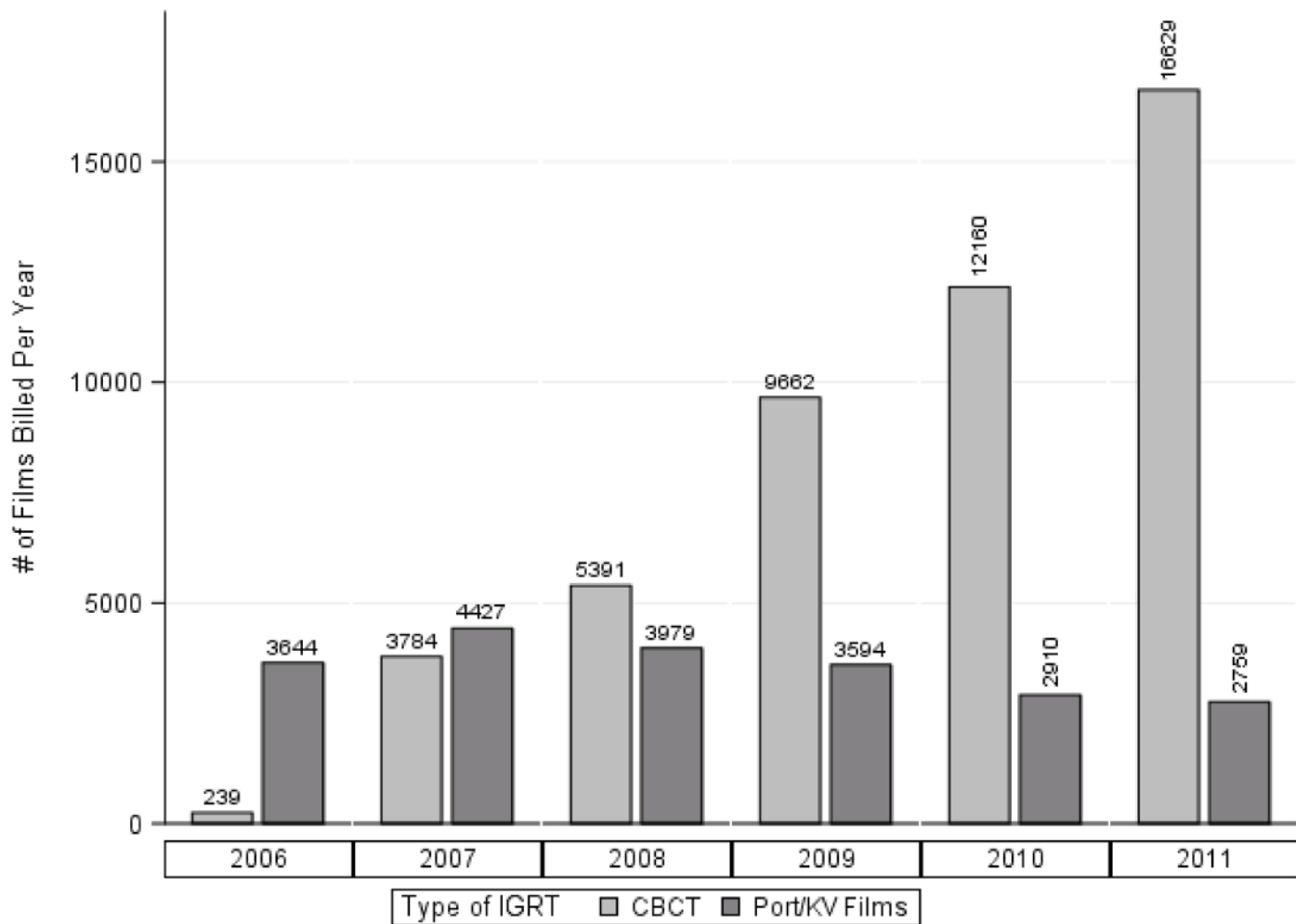

Supplement: S2 Fig — Total number of Medicare claims for CBCT and Port/KV based image guidance in our cohort over time. CBCT rapidly overtook Port/KV imaging as the preferred method of image guidance. (PDF) [file pone.0252053.s002.pdf]

Distribution of CBCT Utilization in Patients Who Recieved hfIGRT

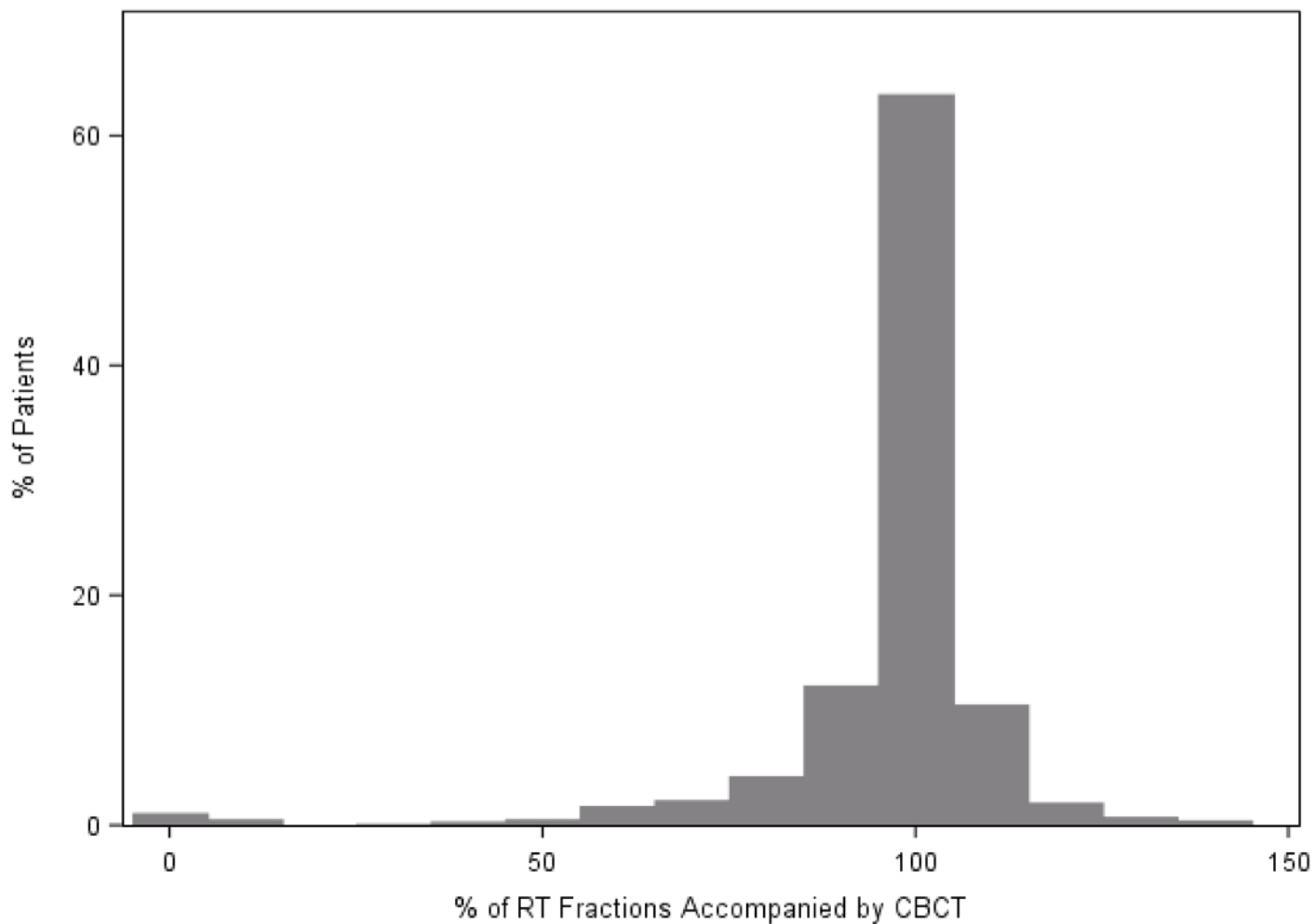

Supplement: S3 Fig — Percent of radiation fractions accompanied by CBCT in patient who are receiving hfIGRT. Clearly the vast majority of patients who are receiving hfIGRT are getting most of their fractions with CBCT imaging. (PDF) [file pone.0252053.s003.pdf]

IGRT Cost by LCD

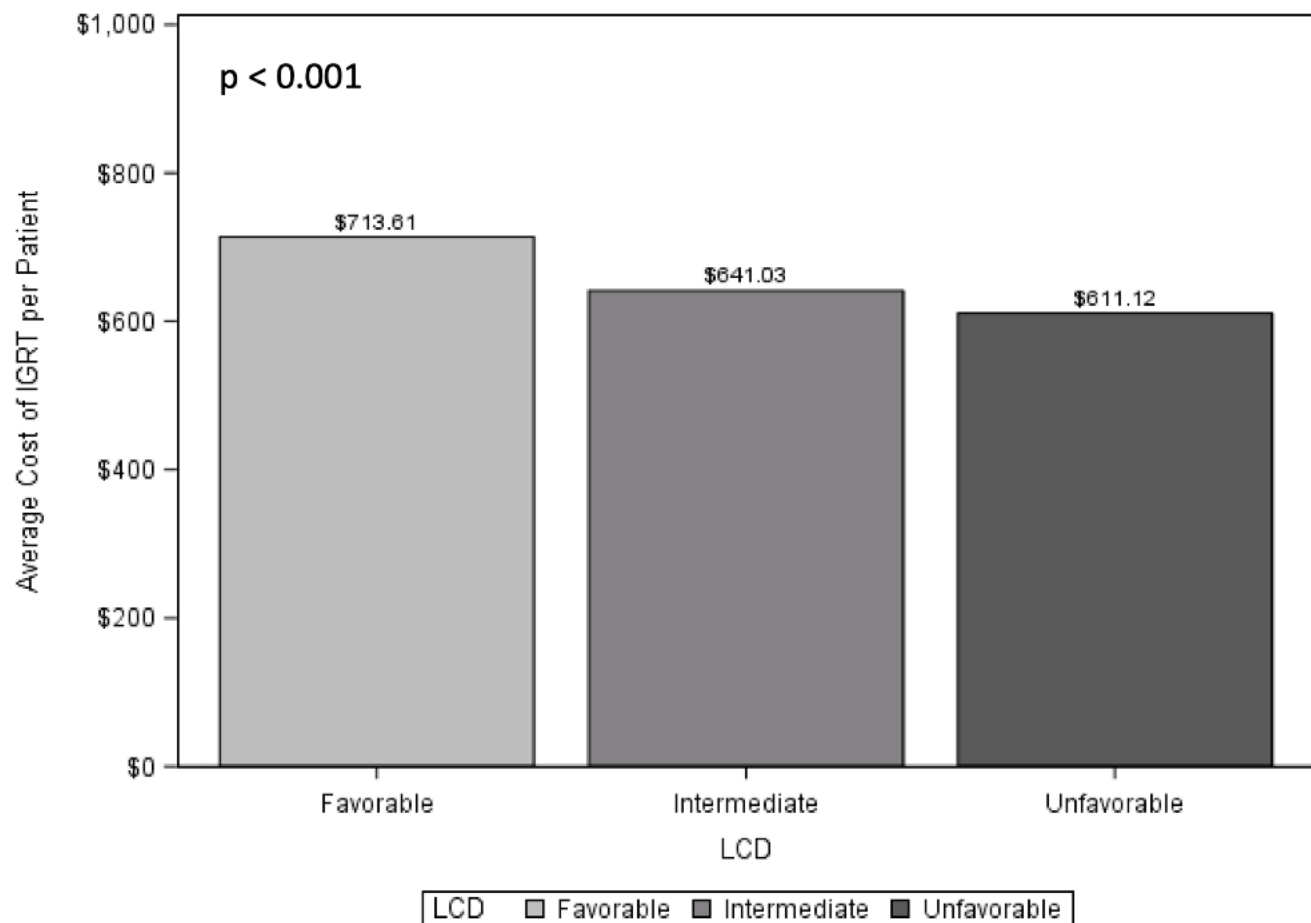

Supplement: S5 Fig — Shows the average cost of image guidance in IMRT favorable, intermediate, and unfavorable regions of the united states. Costs were calculated for each patient by summing the Medicare reimbursement for all image guidance associated bills. (PDF) [file pone.0252053.s005.pdf]
