# Supplementary material for: Efficacy and cost of high-frequency IGRT in elderly stage III non-small-cell lung cancer patients
Source: PLoS One. 2021 May 27;16(5):e0252053. doi: 10.1371/journal.pone.0252053 (PMC8158910; doi:10.1371/journal.pone.0252053)
Supplement: S4 Fig — (A) Histogram showing the distribution of IGRT utilization by physicians. The x-axis shows the percentage of stage III NSCLC patients each radiation oncologist uses hfIGRT in. The Y axis represent the percentage of radiation oncologist who use hfIGRT with that frequency. (B) The same as S4a Fig, except excluding radiation oncologist who used hfIGRT in none of their patients. (PDF) [file pone.0252053.s004.pdf]

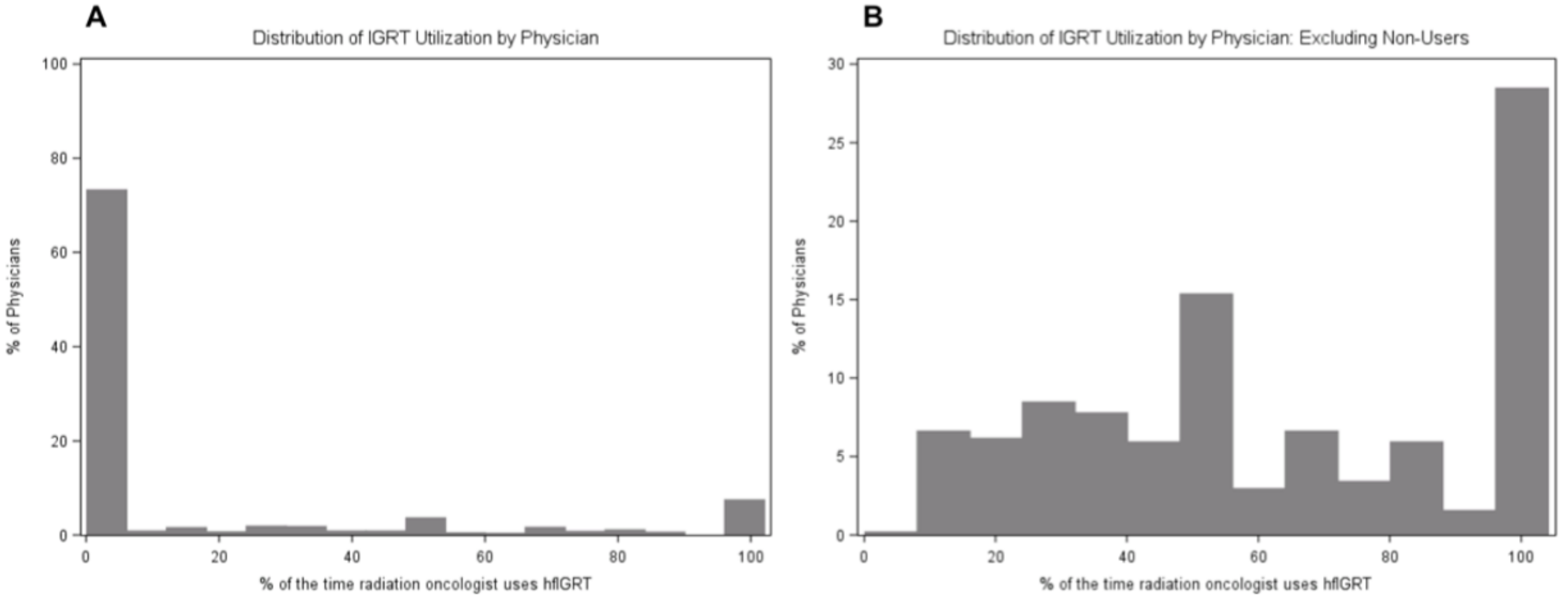
A
Distribution of IGRT Utilization by Physician
% of Physicians
% of the time radiation oncologist uses hfIGRT
0
20
40
60
80
100
B
Distribution of IGRT Utilization by Physician: Excluding Non-Users
0
5
10
15
20
25
30
