# Supplementary material for: Efficacy and cost of high-frequency IGRT in elderly stage III non-small-cell lung cancer patients
Source: PLoS One. 2021 May 27;16(5):e0252053. doi: 10.1371/journal.pone.0252053 (PMC8158910; doi:10.1371/journal.pone.0252053)
Supplement: S1 Table — Consort diagram showing selection criteria and number of patients left in cohort after each selection criteria is applied. (DOCX) [file pone.0252053.s006.docx]

| **Selection Criteria** | **Number of Remaining Obs.** | |
| --- | --- | --- |
|  | **hfIGRT** | **No hfIGRT** |
| All Lung Cancer Patients in the SEER-Medicare Database | 600,828 | |
| Only Select SEER-Records for First Cancer Diagnosis | 548,939 | |
| 1st Cancer is of the Lung | 471,916 | |
| Reporting Source should not be autopsy or death certificate. | 459,716 | |
| Age of diagnosis should be greater than 65. | 381,444 | |
| Original or current reason for entitlement should be age. | 379,047 | |
| Delete if date of death between SEER and Medicare if off by > 3 months. | 378,158 | |
| Take only cases diagnosed from 2006 to 2011 | 141,552 | |
| Exclude members of an HMO 12 months before to 12 months after diagnosis. | 102,101 | |
| Have both Part A & B Coverage | 89,680 | |
| Non-small cell lung cancer | 64,949 | |
| Stage III | 15,874 | |
| Definitive Treatment: 25 to 45 Radiation Fractions | 5,157 | |
| Cohort | 962 | 3468 |
