# Supplementary material for: Efficacy and cost of high-frequency IGRT in elderly stage III non-small-cell lung cancer patients
Source: PLoS One. 2021 May 27;16(5):e0252053. doi: 10.1371/journal.pone.0252053 (PMC8158910; doi:10.1371/journal.pone.0252053)
Supplement: S2 Table — (DOCX) [file pone.0252053.s007.docx]

|  | | | | |
| --- | --- | --- | --- | --- |
| **Treatment** | **ICD-9 Codes** | **CPT/HCPCS Codes** | **Revenue Center/**  **Diagnosis Related Codes** | **DME File MTUSIND Code** |
| Chemotherapy | 99.25,  V58.1, V66.2, V67.2  E9331, E9307 | Q0083, Q0084, Q0085,  96400-96599,  J9000-J9999  G0355-G0362 | 0331, 0332, 0335  410 |  |
| Cone-beam CT (CBCT) |  | 77014, 77421 |  |  |
| Portal/KV Films |  | 77417 |  |  |
| Image-guided Radiation Therapy (IGRT = CBCT + Port/KV) |  | 77014, 77421, 77417 |  |  |
| Intensity-modulate Radiation Therapy (IMRT) |  | 77301, 77338, 77418, G0174, G0178 |  |  |
| PET staging scan |  | 78810-78816  G0030-G0047  G0210-G0235  G0125-G0126  G0163-G0165  G0252-G0254  G0296, G0330, G0331, G0336 |  |  |
| Radiation | V58.0, V66.1, V67.1  92.21,92.22,92.23,92.24,92.25,92.26,92.27,92.28,92.29 | 77401-77499,  77520-77525,  77750-77799 | 0330, 0333 |  |
| Supplemental O2 |  |  |  | 4 |
| Surgery | 321, 323, 324, 325, 326, 3220, 3229, 3230, 3239, 3241, 3249, 3250, 3259 | 32440, 32442, 32445, 32480, 32482, 32484, 32486, 32488, 32500, 32503, 32504, 32520, 32522, 32525, 32657, 32663 |  |  |
| Abbreviations: ICD-9, International Classification of Diseases, 9th Revision, Clinical Modification (ICD-9-CM); CPT/HCPCS, Current Procedural Terminology/ Healthcare Common Procedure Coding System | | | | |
