# Supplementary material for: Efficacy and cost of high-frequency IGRT in elderly stage III non-small-cell lung cancer patients
Source: PLoS One. 2021 May 27;16(5):e0252053. doi: 10.1371/journal.pone.0252053 (PMC8158910; doi:10.1371/journal.pone.0252053)
Supplement: S3 Table — (DOCX) [file pone.0252053.s008.docx]

|  | | | | | | | | | | |
| --- | --- | --- | --- | --- | --- | --- | --- | --- | --- | --- |
| Characteristic | ***Unmatched*** | | | | | ***Matched*** | | | | |
|  | IGRT (N) | IGRT (%) | No IGRT (N) | No IGRT (%) | P-value | IGRT (N) | IGRT (%) | No IGRT (N) | No IGRT (%) | P-value |
| Age |  |  |  |  | 0.41 |  |  |  |  | 0.80 |
| 65 - 74 | 508 | 52.8 | 1911 | 55.1 |  | 364 | 51.1 | 376 | 52.7 |  |
| 75 - 84 | 397 | 41.3 | 1349 | 38.9 |  | 306 | 42.9 | 294 | 41.2 |  |
| 85+ | 57 | 5.9 | 208 | 6 |  | 43 | 6 | 43 | 6 |  |
| Sex |  |  |  |  | 0.89 |  |  |  |  | 0.29 |
| Male | 528 | 54.9 | 1895 | 54.6 |  | 394 | 55.3 | 374 | 52.5 |  |
| Female | 434 | 45.1 | 1573 | 45.4 |  | 319 | 44.7 | 339 | 47.5 |  |
| Race |  |  |  |  | 0.07 |  |  |  |  | 0.05 |
| White | ≥838 | ≥87.2 | 2979 | 85.9 |  | ≥632 | ≥88.3 | ≥619 | ≥86.8 |  |
| Black | 77 | 8 | 308 | 8.9 |  | 51 | 7.2 | 54 | 7.6 |  |
| Hispanic | ≤11 | ≤1.1 | 33 | 1 |  | ≤11 | ≤1.5 | ≤11 | ≤1.5 |  |
| Other | 35 | 3.6 | 148 | 4.3 |  | 19 | 2.7 | 29 | 4.1 |  |
| State |  |  |  |  | <.01 |  |  |  |  | 0.11 |
| California | ≥178 | ≥18.6 | 777 | 22.4 |  | ≥100 | ≥15.4 | ≥146 | ≥21.3 |  |
| Connecticut | 54 | 5.6 | 254 | 7.3 |  | 54 | 7.6 | 42 | 5.9 |  |
| Georgia | 145 | 15.1 | 507 | 14.6 |  | 93 | 13.0 | 108 | 15.1 |  |
| Hawaii | ≤11 | ≤1.1 | 45 | 1.3 |  | ≤11 | ≤1.5 | ≤11 | ≤1.5 |  |
| Iowa | 76 | 7.9 | 228 | 6.6 |  | 75 | 10.5 | 50 | 7 |  |
| Kentucky | 101 | 10.5 | 368 | 10.6 |  | 76 | 10.7 | 68 | 9.5 |  |
| Louisiana | 70 | 7.3 | 285 | 8.2 |  | 63 | 8.8 | 53 | 7.4 |  |
| Michigan | 83 | 8.6 | 264 | 7.6 |  | 80 | 11.2 | 71 | 10 |  |
| New Jersey | 142 | 14.8 | 459 | 13.2 |  | 81 | 11.4 | 81 | 11.4 |  |
| New Mexico | ≤11 | ≤1.1 | 63 | 1.8 |  | ≤11 | ≤1.5 | ≤11 | ≤1.5 |  |
| Utah | ≤11 | ≤1.1 | 30 | 0.9 |  | ≤11 | ≤1.5 | ≤11 | ≤1.5 |  |
| Washington | 80 | 8.3 | 188 | 5.4 |  | 58 | 8.1 | 61 | 8.6 |  |
| Local Coverage Determination |  |  |  |  | 0.62 |  |  |  |  | 0.48 |
| Favorable | 220 | 22.9 | 750 | 21.6 |  | 170 | 23.8 | 177 | 24.8 |  |
| Intermediate | 562 | 58.4 | 2033 | 58.6 |  | 395 | 55.4 | 406 | 56.9 |  |
| Unfavorable | 180 | 18.7 | 685 | 19.8 |  | 148 | 20.8 | 130 | 18.2 |  |
| Year of Diagnosis |  |  |  |  | <.01 |  |  |  |  | 0.85 |
| 2006 | 14 | 1.5 | 785 | 22.6 |  | 14 | 2 | 12 | 1.7 |  |
| 2007 | 71 | 7.4 | 741 | 21.4 |  | 71 | 10 | 73 | 10.2 |  |
| 2008 | 103 | 10.7 | 663 | 19.1 |  | 101 | 14.2 | 105 | 14.7 |  |
| 2009 | 202 | 21 | 534 | 15.4 |  | 157 | 22 | 142 | 19.9 |  |
| 2010 | 241 | 25.1 | 402 | 11.6 |  | 175 | 24.5 | 193 | 27.1 |  |
| 2011 | 331 | 34.4 | 343 | 9.9 |  | 195 | 27.3 | 188 | 26.4 |  |
| Charlson Score (No COPD) |  |  |  |  | 0.16 |  |  |  |  | 0.44 |
| 0 | ≥503 | ≥52.3 | 1952 | 56.3 |  | ≥372 | ≥52.2 | ≥397 | ≥55.7 |  |
| 1-2 | 359 | 37.3 | 1199 | 34.6 |  | 267 | 37.4 | 245 | 34.4 |  |
| > 2 | 95 | 9.9 | 317 | 9.1 |  | 67 | 9.4 | 66 | 9.3 |  |
| COPD | 457 | 47.5 | 1590 | 45.8 | 0.36 | 329 | 46.1 | 330 | 46.3 | 0.96 |
| Supplemental O2 | 234 | 24.3 | 864 | 24.9 | 0.71 | 170 | 23.8 | 176 | 24.7 | 0.71 |
| Homebound | ≤11 | ≤1.1 | 67 | 1.9 | <.01 | ≤11 | ≤1.5 | ≤11 | ≤1.5 | 0.53 |
| Histology |  |  |  |  | <.01 |  |  |  |  | 0.18 |
| Adenocarcinoma | 377 | 39.2 | 1086 | 31.3 |  | 276 | 38.7 | 237 | 33.2 |  |
| SCC | 397 | 41.3 | 1463 | 42.2 |  | 295 | 41.4 | 314 | 44 |  |
| Large Cell | 23 | 2.4 | 114 | 3.3 |  | 19 | 2.7 | 21 | 2.9 |  |
| Other | 165 | 17.2 | 805 | 23.2 |  | 123 | 17.3 | 141 | 19.8 |  |
| Stage |  |  |  |  | 0.37 |  |  |  |  | 0.53 |
| Stage IIIA | 489 | 50.8 | 1706 | 49.2 |  | 366 | 51.3 | 354 | 49.6 |  |
| Stage IIIB | 473 | 49.2 | 1762 | 50.8 |  | 347 | 48.7 | 359 | 50.4 |  |
| T-Stage |  |  |  |  | 0.74 |  |  |  |  | 0.97 |
| TX | 47 | 4.9 | 148 | 4.3 |  | 33 | 4.6 | 30 | 4.2 |  |
| T0 | ≤11 | ≤1.1 | 18 | 0.5 |  | ≤11 | ≤1.5 | ≤11 | ≤1.5 |  |
| T1 | 132 | 13.7 | 471 | 13.6 |  | 101 | 14.2 | 108 | 15.1 |  |
| T2 | 326 | 33.9 | 1135 | 32.7 |  | 235 | 33.0 | 227 | 31.8 |  |
| T3 | 96 | 10 | 327 | 9.4 |  | 72 | 10.1 | 70 | 9.8 |  |
| T4 | ≥350 | ≥36.4 | 1369 | 39.5 |  | ≥261 | ≥36.6 | ≥267 | ≥37.5 |  |
| Tumor Size |  |  |  |  | 0.54 |  |  |  |  | 0.95 |
| < 2.0 | 62 | 6.4 | 246 | 7.1 |  | 42 | 5.9 | 45 | 6.3 |  |
| 2.0-5.0 | 435 | 45.2 | 1589 | 45.8 |  | 329 | 46.1 | 319 | 44.7 |  |
| > 5.0 | 328 | 34.1 | 1105 | 31.9 |  | 245 | 34.4 | 249 | 34.9 |  |
| Unknown | 137 | 14.2 | 528 | 15.2 |  | 97 | 13.6 | 100 | 14 |  |
| Tumor Laterality |  |  |  |  | 0.83 |  |  |  |  | 0.51 |
| Right | ≥556 | ≥57.8 | ≥2007 | ≥57.8 |  | ≥403 | ≥57.2 | ≥387 | ≥54.8 |  |
| Left | 383 | 39.8 | 1416 | 40.8 |  | 288 | 40.4 | 304 | 42.6 |  |
| Unpaired | ≤11 | ≤1.1 | ≤11 | 0.3 |  | ≤11 | ≤1.5 | ≤11 | ≤1.5 |  |
| Unknown | 12 | 1.2 | 34 | 1 |  | ≤11 | ≤1.5 | ≤11 | ≤1.5 |  |
| Tumor Location |  |  |  |  | 0.15 |  |  |  |  | 0.08 |
| Main bronchus | 52 | 5.4 | 223 | 6.4 |  | 35 | 4.9 | 56 | 7.9 |  |
| Upper lobe | ≥573 | ≥59.6 | 2023 | 58.3 |  | ≥422 | ≥59.2 | ≥420 | ≥58.9 |  |
| Middle lobe | 27 | 2.8 | 138 | 4 |  | 20 | 2.8 | 19 | 2.7 |  |
| Lower lobe | 243 | 25.3 | 872 | 25.1 |  | 183 | 25.7 | 173 | 24.3 |  |
| Lung NOS | 56 | 5.8 | 182 | 5.2 |  | 42 | 5.9 | 34 | 4.8 |  |
| Other | ≤11 | ≤1.1 | 30 | 0.9 |  | ≤11 | ≤1.5 | ≤11 | ≤1.5 |  |
| PET Staging | 902 | 93.8 | 3194 | 92.1 | 0.08 | 664 | 93.1 | 659 | 92.4 | 0.61 |
| # of nodes positive |  |  |  |  | 0.63 |  |  |  |  | 0.81 |
| 0 | 40 | 4.2 | 167 | 4.8 |  | 26 | 3.6 | 22 | 3.1 |  |
| 1-3 | 126 | 13.1 | 432 | 12.5 |  | 85 | 11.9 | 79 | 11.1 |  |
| 4+ | 25 | 2.6 | 109 | 3.1 |  | 18 | 2.5 | 22 | 3.1 |  |
| Unknown | 771 | 80.1 | 2760 | 79.6 |  | 584 | 81.9 | 590 | 82.7 |  |
| Treatment Type |  |  |  |  | 0.23 |  |  |  |  | 0.45 |
| Trimodality | 71 | 7.4 | 289 | 8.3 |  | 54 | 7.6 | 52 | 7.3 |  |
| Chemotherapy & radiation | 735 | 76.4 | 2534 | 73.1 |  | 530 | 74.3 | ≥550 | ≥77.2 |  |
| Surgery & radiation | 16 | 1.7 | 65 | 1.9 |  | 14 | 2 | ≤11 | ≤1.5 |  |
| Radiation alone | 140 | 14.6 | 580 | 16.7 |  | 115 | 16.1 | 101 | 14 |  |
| # of RT Fractions |  |  |  |  | <.01 |  |  |  |  | 0.92 |
| 25 - 29 | 196 | 20.4 | 678 | 19.6 |  | 141 | 19.8 | 137 | 19.2 |  |
| 30 - 34 | 361 | 37.5 | 1558 | 44.9 |  | 280 | 39.3 | 277 | 38.8 |  |
| 35 - 40 | 405 | 42.1 | 1232 | 35.5 |  | 292 | 41 | 299 | 41.9 |  |
| IMRT | 531 | 55.2 | 487 | 14 | <0.01 | 298 | 41.8 | 308 | 43.2 | 0.59 |
| Type of Treatment Center |  |  |  |  | <.01 |  |  |  |  | 0.25 |
| Free Standing | 401 | 41.7 | 1092 | 31.5 |  | 261 | 36.6 | 290 | 40.7 |  |
| Hospital Based | ≥550 | ≥57.2 | 2350 | 67.8 |  | ≥441 | ≥61.9 | ≥412 | ≥57.8 |  |
| Both | ≤11 | ≤1.1 | 26 | 0.7 |  | ≤11 | ≤1.5 | ≤11 | ≤1.5 |  |
| Rural vs. Urban |  |  |  |  | 0.06 |  |  |  |  | 0.50 |
| Rural | 161 | 16.7 | 674 | 19.4 |  | 141 | 19.8 | 131 | 18.4 |  |
| Urban | 801 | 83.3 | 2794 | 80.6 |  | 572 | 80.2 | 582 | 81.6 |  |
| Radiation Oncologist Density |  |  |  |  | <.01 |  |  |  |  | 0.31 |
| 1st quartile | 285 | 29.6 | 1131 | 32.6 |  | ≥201 | ≥28.2 | ≥197 | ≥27.6 |  |
| 2nd quartile | ≥287 | ≥29.9 | 893 | 25.7 |  | 194 | 27.2 | 226 | 31.8 |  |
| 3rd quartile | 221 | 23 | 788 | 22.7 |  | 188 | 26.4 | 176 | 24.7 |  |
| 4th quartile | 158 | 16.4 | 611 | 17.6 |  | 119 | 16.7 | 103 | 14.4 |  |
| Unknown | ≤11 | ≤1.1 | 45 | 1.3 |  | ≤11 | ≤1.5 | ≤11 | ≤1.5 |  |
| General Surgeon Density |  |  |  |  | <.01 |  |  |  |  | 0.21 |
| 1st quartile | 252 | 26.2 | 1095 | 31.6 |  | 194 | 27.3 | 198 | 27.8 |  |
| 2nd quartile | 234 | 24.3 | 804 | 23.2 |  | 155 | 21.7 | 178 | 25 |  |
| 3rd quartile | ≥248 | ≥26.2 | 821 | 23.7 |  | ≥192 | ≥26.9 | ≥199 | ≥28 |  |
| 4th quartile | 217 | 22.6 | 703 | 20.3 |  | 161 | 22.6 | 127 | 17.8 |  |
| Unknown | ≤11 | ≤1.1 | 45 | 1.3 |  | ≤11 | ≤1.5 | ≤11 | ≤1.5 |  |
| Physician Experience |  |  |  |  | <.01 |  |  |  |  | 0.13 |
| 1st quartile | 173 | 18 | ≥928 | ≥26.7 |  | 145 | 20.3 | 150 | 21 |  |
| 2nd quartile | 244 | 25.4 | 861 | 24.8 |  | 184 | 25.8 | 216 | 30.3 |  |
| 3rd quartile | 335 | 34.8 | 772 | 22.3 |  | 217 | 30.4 | 183 | 25.7 |  |
| 4th quartile | 210 | 21.8 | 896 | 25.8 |  | 167 | 23.4 | 164 | 23 |  |
| Other | 0 | 0 | ≥11 | 0.3 |  | 0 | 0 | 0 | 0 |  |
| * To protect patient anonymity, all cells with values between 1 and 11 were masked with the indicator “≤11.” Then the largest cell in the same column for the same characteristic was adjusted in the opposite direction so that the total number of observations in that column remained the same. | | | | | | | | | | |
