# Supplementary material for: Efficacy and cost of high-frequency IGRT in elderly stage III non-small-cell lung cancer patients
Source: PLoS One. 2021 May 27;16(5):e0252053. doi: 10.1371/journal.pone.0252053 (PMC8158910; doi:10.1371/journal.pone.0252053)
Supplement: S4 Table — (DOCX) [file pone.0252053.s009.docx]

|  | | | | |
| --- | --- | --- | --- | --- |
|  | | | | |
| **Pulmonary Complications:** | | | | |
| **Narrow Acute Lung Toxicity** | | | |  |
|  | ICD-9 | | 508.0 | Acute pulmonary manifestations due to radiation |
| **Broad Acute Long Toxicity** | | | |  |
|  | ICD-9 | | 508.0 | Acute pulmonary manifestations due to radiation |
|  |  | | 485 | Bronchopneumonia, organism unspecified |
|  |  | | 486 | Pneumonia, organism unspecified |
|  |  | | 482.9 | Bacterial pneumonia, unspecified |
|  |  | | 514 | Pulmonary congestion and hypostasis (NOT attributable to heart failure) |
|  | | | |  |
| **Esophageal Complications:** | | | | |
| **Esophagitis and Dysphagia** | | | |  |
|  | ICD-9 | 530.1 | | Esophagitis |
|  |  | 530.11 | | Esophagitis, unspecified |
|  |  | 530.12 | | Esophagitis, acute |
|  |  | 530.19 | | Esophagitis, other types |
|  |  | 787.2 | | Dysphagia |
|  |  | 787.20 | | Dysphagia unspecified |
|  |  | 787.21 | | Dysphagia oral phase |
|  |  | 787.22 | | Dysphagia oropharyngeal phase |
|  |  | 787.23 | | Dysphagia pharyngeal phase |
|  |  | 787.24 | | Dysphagia pharyngoesophageal phase |
|  |  | 787.29 | | Other dysphagia |
|  |  | 784.1 | | Throat pain |
| **Dehydration** | | | |  |
|  | ICD-9 | 276.5 | | Volume depletion |
|  |  | 276.50 | | Volume depletion, unspecified |
|  |  | 276.51 | | Dehydration |
|  |  | 276.52 | | Hypovolemia |
| **Mucositis** | |  | |  |
|  | ICD-9 | 528.0 | | Stomatitis and mucositis |
|  |  | 528.00 | | Mucositis, unspecified |
|  |  | 528.01 | | Mucositis (ulcerative) due to antineoplastic therapy |
|  |  | 528.02 | | Mucositis (ulcerative) due to other drug |
|  |  | 528.09 | | Mucositis (ulcerative) due to other cause |
|  |  | 538 | | Gastrointestinal mucositis |
| **Feeding Tube Placement** | | | |  |
|  | ICD-9 | 43.1 | | Gastrostomy |
|  |  | 43.11 | | Percutaneous enterogastrostomy |
|  |  | 43.19 | | Other gastrostomy |
|  |  | 46.3 | | Other enterostomy |
|  |  | 46.31 | | Delayed opening of other enterostomy |
|  |  | 46.32 | | Percutanoues enterojejunostomy |
|  |  | 46.39 | | Feeding enterostomy or Duodenostomy |
|  | CPT | 43246 | | Upper GI endoscopy with directed placement of percutaneous gastrostomy tube |
|  |  | 43750 | | Percutaneous placement of gastrostomy tube |
|  |  | 43760 | | Change of gastrostomy tube |
|  |  | 43761 | | Repositioning of gastric feedint tube through the duodenum for enteric nutrition |
|  |  | 74350 | | Percutaneous placement of gastrostomy tube, radiological supervision/interpretation |
|  |  | 74355 | | Percutaneous placement of enteroclysis tube, radiological supervision/interpretation |
|  |  | B4086 | | Gastrostomy/jejunostomy tube, any material |
|  |  | 44373 | | Small intestinal endoscopy with conversion of PEG tube to PEJ tube |
|  |  | 44372 | | Small intestinal endoscopy with placement of PEJ tube |
|  |  | 44015 | | Tube or needle catheter jejunostomy for enteral alimentation |
|  |  | 44201 | | Surgical laparoscopy with jejunostomy for feeding or nutrition |
| Abbreviations: ICD-9, International Classification of Diseases, 9th Revision, Clinical Modification (ICD-9-CM); CPT, Current Procedural Terminology/ Healthcare Common Procedure Coding System | | | | |
