# Supplementary material for: Efficacy and cost of high-frequency IGRT in elderly stage III non-small-cell lung cancer patients
Source: PLoS One. 2021 May 27;16(5):e0252053. doi: 10.1371/journal.pone.0252053 (PMC8158910; doi:10.1371/journal.pone.0252053)
Supplement: S5 Table — (DOCX) [file pone.0252053.s010.docx]

|  | | | |
| --- | --- | --- | --- |
| Predictor | Odds Ratio | 95% CI | P-value |
| Year of Diagnosis |  |  |  |
| 2006 | 1 (Ref) |  |  |
| 2007 | 6.02 | 3.32 - 10.91 | <.01 |
| 2008 | 8.35 | 4.66 - 14.95 | 0.03 |
| 2009 | 18.37 | 10.40 - 32.45 | <.01 |
| 2010 | 31.72 | 17.98 - 55.99 | <.01 |
| 2011 | 50.18 | 28.47 - 88.44 | <.01 |
| LCD |  |  |  |
| Favorable | 1 (Ref) |  |  |
| Intermediate | 1.05 | 0.83 - 1.34 | <.01 |
| Unfavorable | 0.66 | 0.50 - 0.88 | <.01 |
| General Surgeon Density |  |  |  |
| 1st quartile | 1 (Ref) |  |  |
| 2nd quartile | 1.08 | 0.81 - 1.46 | 0.02 |
| 3rd quartile | 1.53 | 1.12 - 2.09 | 0.09 |
| 4th quartile | 1.82 | 1.29 - 2.58 | <.01 |
| Unknown | - | - | - |
| Radiation Oncologist Density |  |  |  |
| 1st quartile | 1 (Ref) |  |  |
| 2nd quartile | 0.84 | 0.63 - 1.14 | <.01 |
| 3rd quartile | 0.66 | 0.48 - 0.91 | 0.12 |
| 4th quartile | 0.67 | 0.46 - 0.96 | 0.14 |
| Physician Experience |  |  |  |
| 1st quartile | 1 (Ref) |  |  |
| 2nd quartile | 2.12 | 1.64 - 2.74 | 0.33 |
| 3rd quartile | 3.45 | 2.68 - 4.44 | <.01 |
| 4th quartile | 2.06 | 1.57 - 2.70 | 0.60 |
| Type of Treatment Center |  |  |  |
| Free Standing | 1 (Ref) |  |  |
| Hospital Based | 0.63 | 0.53 - 0.76 | 0.18 |
| Both | 0.13 | 0.03 - 0.66 | 0.03 |
| # of Radiation Fractions |  |  |  |
| 25 - 29 | 1 (Ref) |  |  |
| 30 - 34 | 0.68 | 0.54 - 0.86 | <.01 |
| 35 - 40 | 0.93 | 0.74 - 1.18 | 0.19 |
| IMRT | 6.47 | 5.38 - 7.80 | <.01 |
