# Supplementary material for: Efficacy and cost of high-frequency IGRT in elderly stage III non-small-cell lung cancer patients
Source: PLoS One. 2021 May 27;16(5):e0252053. doi: 10.1371/journal.pone.0252053 (PMC8158910; doi:10.1371/journal.pone.0252053)
Supplement: S6 Table — (DOCX) [file pone.0252053.s011.docx]

|  | | |
| --- | --- | --- |
| Parameter | Univariate  HR (95% CI, P-value) | Multivariate  HR (95% CI, P-Value) |
| Daily IGRT |  |  |
| No | Reference | Reference |
| Yes | 0.96 (0.86 - 1.08, 0.53) | 0.97 (0.86 - 1.09, 0.60) |
| Age |  |  |
| 65 - 74 | Reference | Reference |
| 75 - 84 | 1.09 (0.98 - 1.20, 0.10) | 1.05 (0.95 - 1.17, 0.31) |
| 85+ | 1.01 (0.82 - 1.25, 0.90) | 0.99 (0.80 - 1.23, 0.94) |
| Race |  |  |
| White | Reference | Reference |
| Black | 0.98 (0.82 - 1.15, 0.78) | * |
| Hispanic | 0.98 (0.57 - 1.69, 0.94) | * |
| Other | 0.98 (0.77 - 1.24, 0.85) | * |
| COPD |  |  |
| No | Reference | Reference |
| Yes | 1.42 (1.29 - 1.56, <.01) | 1.26 (1.13 - 1.39, <.01) |
| Charlson Score (no COPD) |  |  |
| 0 | Reference | Reference |
| 1-2 | 1.22 (1.10 - 1.35, <.01) | 1.14 (1.02 - 1.26, 0.02) |
| > 2 | 1.56 (1.34 - 1.82, <.01) | 1.41 (1.21 - 1.66, <.01) |
| Supplemental O2 |  |  |
| No | Reference | Reference |
| Yes | 1.48 (1.33 - 1.64, <.01) | 1.21 (1.08 - 1.35, <.01) |
| Homebound |  |  |
| No | Reference | Reference |
| Yes | 1.82 (1.33 - 2.49, <.01) | 1.39 (1.01 - 1.92, 0.04) |
| Stage |  |  |
| Stage IIIA | Reference | Reference |
| Stage IIIB | 1.14 (1.03 - 1.25, <.01) | 1.08 (0.98 - 1.19, 0.11) |
| T-Stage |  |  |
| TX | Reference | Reference |
| T0 | 0.65 (0.30 - 1.41, 0.27) | * |
| T1 | 0.72 (0.56 - 0.93, 0.01) | * |
| T2 | 0.88 (0.70 - 1.11, 0.28) | * |
| T3 | 1.03 (0.80 - 1.34, 0.80) | * |
| T4 | 0.98 (0.78 - 1.23, 0.85) | * |
| Tumor Size |  |  |
| < 2.0 | Reference | Reference |
| 2.0-5.0 | 1.15 (0.94 - 1.41, 0.17) | 1.11 (0.90 - 1.37, 0.32) |
| > 5.0 | 1.28 (1.04 - 1.57, 0.02) | 1.21 (0.98 - 1.50, 0.07) |
| Unknown | 1.52 (1.22 - 1.90, <.01) | 1.38 (1.10 - 1.74, <.01) |
| Histology |  |  |
| Adenocarcinoma | Reference | Reference |
| SCC | 1.26 (1.13 - 1.41, <.01) | 1.14 (1.02 - 1.28, 0.02) |
| Large Cell | 1.41 (1.09 - 1.82, <.01) | 1.28 (0.99 - 1.66, 0.06) |
| Other | 0.99 (0.86 - 1.13, 0.83) | 0.90 (0.79 - 1.03, 0.14) |
| Laterality |  |  |
| Right | Reference | Reference |
| Left | 0.96 (0.87 - 1.05, 0.37) | * |
| Unpaired | 0.77 (0.19 - 3.09, 0.72) | * |
| Unknown | 0.79 (0.47 - 1.32, 0.37) | * |
| Tumor Location |  |  |
| Main bronchus | Reference | Reference |
| Upper lobe | 0.73 (0.60 - 0.89, <.01) | 0.87 (0.72 - 1.07, 0.18) |
| Middle lobe | 0.87 (0.64 - 1.17, 0.35) | 1.02 (0.75 - 1.39, 0.90) |
| Lower lobe | 1.09 (0.89 - 1.33, 0.42) | 1.26 (1.02 - 1.55, 0.03) |
| Lung NOS | 0.84 (0.64 - 1.11, 0.23) | 0.85 (0.64 - 1.12, 0.25) |
| Other | 1.47 (0.90 - 2.42, 0.13) | 1.86 (1.12 - 3.07, 0.02) |
| PET |  |  |
| No | Reference | Reference |
| Yes | 1.00 (0.83 - 1.20, 1.00) | * |
| # of Positive Nodes |  |  |
| 0 | Reference | Reference |
| 1-3 | 0.85 (0.65 - 1.11, 0.23) | * |
| 4+ | 0.77 (0.52 - 1.12, 0.17) | * |
| Unknown | 1.16 (0.92 - 1.46, 0.20) | * |
| Treatment Type |  |  |
| Trimodality | Reference | Reference |
| Chemotherapy & radiation | 1.40 (1.15 - 1.70, <.01) | 1.54 (1.25 - 1.90, <.01) |
| Surgery & radiation | 1.06 (0.69 - 1.63, 0.78) | 1.03 (0.67 - 1.58, 0.90) |
| Radiation alone | 1.54 (1.24 - 1.92, <.01) | 1.52 (1.20 - 1.93, <.01) |
| # of RT Fractions |  |  |
| 25 - 29 | Reference | Reference |
| 30 - 34 | 0.74 (0.65 - 0.84, <.01) | 0.66 (0.58 - 0.75, <.01) |
| 35 - 40 | 0.72 (0.64 - 0.82, <.01) | 0.64 (0.56 - 0.73, <.01) |
| Type of Treatment Center |  |  |
| Free Standing | Reference | Reference |
| Hospital Based | 1.00 (0.90 - 1.10, 0.96) | * |
| Both | 1.37 (0.80 - 2.32, 0.25) | * |
| Rural vs. Urban |  |  |
| Rural | Reference | Reference |
| Urban | 0.93 (0.83 - 1.05, 0.27) | * |
| Radiation Oncologist Density |  |  |
| 1st quartile | Reference | Reference |
| 2nd quartile | 0.98 (0.87 - 1.11, 0.73) | * |
| 3rd quartile | 0.99 (0.88 - 1.13, 0.93) | * |
| 4th quartile | 0.90 (0.78 - 1.04, 0.15) | * |
| Unknown | 0.99 (0.62 - 1.58, 0.96) | * |
| General Surgeon Density |  |  |
| 1st quartile | Reference | Reference |
| 2nd quartile | 0.98 (0.86 - 1.11, 0.72) | * |
| 3rd quartile | 1.01 (0.89 - 1.14, 0.91) | * |
| 4th quartile | 1.00 (0.87 - 1.14, 0.96) | * |
| Unknown | 1.01 (0.63 - 1.61, 0.97) | * |
| Physician Experience |  |  |
| 1st quartile | Reference | Reference |
| 2nd quartile | 0.98 (0.86 - 1.12, 0.74) | * |
| 3rd quartile | 0.91 (0.79 - 1.04, 0.15) | * |
| 4th quartile | 0.96 (0.84 - 1.09, 0.50) | * |
| State |  |  |
| California | Reference | Reference |
| Connecticut | 0.89 (0.72 - 1.11, 0.31) | * |
| Georgia | 0.92 (0.78 - 1.09, 0.34) | * |
| Hawaii | 1.06 (0.66 - 1.70, 0.81) | * |
| Iowa | 1.08 (0.88 - 1.33, 0.46) | * |
| Kentucky | 1.08 (0.91 - 1.29, 0.38) | * |
| Louisiana | 1.31 (1.09 - 1.58, <.01) | * |
| Michigan | 1.09 (0.90 - 1.33, 0.37) | * |
| New Jersey | 1.10 (0.93 - 1.29, 0.26) | * |
| New Mexico | 1.03 (0.70 - 1.52, 0.89) | * |
| Utah | 1.70 (1.07 - 2.69, 0.02) | * |
| Washington | 1.01 (0.82 - 1.26, 0.91) | * |
| Year of Diagnosis |  |  |
| 2006 | Reference | Reference |
| 2007 | 0.97 (0.84 - 1.13, 0.72) | * |
| 2008 | 0.90 (0.77 - 1.06, 0.20) | * |
| 2009 | 0.91 (0.78 - 1.06, 0.24) | * |
| 2010 | 0.83 (0.70 - 0.98, 0.03) | * |
| 2011 | 0.86 (0.73 - 1.02, 0.08) | * |
| IMRT |  |  |
| No | Reference | Reference |
| Yes | 1.07 (0.96 - 1.19, 0.25) | * |
| ^X^ Multivariate Cox regressions were performed using stepwise forward and backwards elimination with threshold values of p ≤ 0.20 and p ≤ 0.05, respectively.  * Covariate auto-excluded from model during forward or backward selection.  Abbrev: HR, hazard ratio. CI, confidence interval. | | |
