# Supplementary material for: Efficacy and cost of high-frequency IGRT in elderly stage III non-small-cell lung cancer patients
Source: PLoS One. 2021 May 27;16(5):e0252053. doi: 10.1371/journal.pone.0252053 (PMC8158910; doi:10.1371/journal.pone.0252053)
Supplement: S7 Table — (DOCX) [file pone.0252053.s012.docx]

|  | | |
| --- | --- | --- |
| Parameter | Univariate  HR (95% CI, P-value) | Multivariate  HR (95% CI, P-Value) |
| Daily IGRT |  |  |
| No | Reference | Reference |
| Yes | 0.82 (0.63 - 1.06, 0.13) | 0.81 (0.62 - 1.05, 0.11) |
| Age |  |  |
| 65 - 74 | Reference | Reference |
| 75 - 84 | 1.11 (0.90 - 1.36, 0.35) | 1.04 (0.84 - 1.29, 0.71) |
| 85+ | 1.09 (0.70 - 1.70, 0.70) | 1.11 (0.69 - 1.77, 0.67) |
| Race |  |  |
| White | Reference | Reference |
| Black | 0.83 (0.56 - 1.22, 0.34) | * |
| Hispanic | 0.98 (0.31 - 3.05, 0.97) | * |
| Other | 1.01 (0.61 - 1.68, 0.95) | * |
| COPD |  |  |
| No | Reference | Reference |
| Yes | 1.05 (0.86 - 1.29, 0.63) | * |
| Charlson Score (no COPD) |  |  |
| 0 | Reference | Reference |
| 1-2 | 1.02 (0.82 - 1.26, 0.87) | * |
| > 2 | 1.00 (0.70 - 1.43, 1.00) | * |
| Supplemental O2 |  |  |
| No | Reference | Reference |
| Yes | 0.98 (0.78 - 1.24, 0.88) | 1.01 (0.79 - 1.28, 0.96) |
| Homebound |  |  |
| No | Reference | Reference |
| Yes | 1.28 (0.61 - 2.71, 0.52) | 1.15 (0.54 - 2.47, 0.71) |
| Stage |  |  |
| Stage IIIA | Reference | Reference |
| Stage IIIB | 0.94 (0.77 - 1.16, 0.58) | 0.93 (0.76 - 1.14, 0.48) |
| T-Stage |  |  |
| TX | Reference | Reference |
| T0 | 0.72 (0.17 - 3.05, 0.65) | * |
| T1 | 0.73 (0.44 - 1.20, 0.21) | * |
| T2 | 0.78 (0.49 - 1.22, 0.27) | * |
| T3 | 0.94 (0.56 - 1.56, 0.81) | * |
| T4 | 0.67 (0.42 - 1.05, 0.08) | * |
| Tumor Size |  |  |
| < 2.0 | Reference | Reference |
| 2.0-5.0 | 1.11 (0.73 - 1.68, 0.64) | * |
| > 5.0 | 1.02 (0.66 - 1.57, 0.93) | * |
| Unknown | 1.30 (0.82 - 2.06, 0.26) | * |
| Histology |  |  |
| Adenocarcinoma | Reference | Reference |
| SCC | 1.00 (0.79 - 1.26, 0.99) | * |
| Large Cell | 1.37 (0.80 - 2.33, 0.25) | * |
| Other | 1.09 (0.82 - 1.43, 0.56) | * |
| Laterality |  |  |
| Right | Reference | Reference |
| Left | 0.86 (0.69 - 1.05, 0.14) | * |
| Unpaired | - | * |
| Unknown | 1.15 (0.47 - 2.79, 0.75) | * |
| Tumor Location |  |  |
| Main bronchus | Reference | Reference |
| Upper lobe | 1.31 (0.77 - 2.21, 0.31) | 1.31 (0.77 - 2.22, 0.32) |
| Middle lobe | 2.06 (1.04 - 4.04, 0.04) | 2.00 (1.01 - 3.95, 0.05) |
| Lower lobe | 1.99 (1.16 - 3.39, 0.01) | 1.97 (1.15 - 3.39, 0.01) |
| Lung NOS | 1.42 (0.72 - 2.79, 0.31) | 1.32 (0.67 - 2.61, 0.42) |
| Other | 2.81 (1.02 - 7.74, 0.05) | 3.13 (1.13 - 8.67, 0.03) |
| PET |  |  |
| No | Reference | Reference |
| Yes | 1.89 (1.13 - 3.17, 0.02) | 1.85 (1.10 - 3.11, 0.02) |
| # of Positive Nodes |  |  |
| 0 | Reference | Reference |
| 1-3 | 1.34 (0.74 - 2.43, 0.33) | * |
| 4+ | 1.08 (0.48 - 2.43, 0.85) | * |
| Unknown | 1.35 (0.79 - 2.30, 0.27) | * |
| Treatment Type |  |  |
| Trimodality | Reference | Reference |
| Chemotherapy & radiation | 1.97 (1.22 - 3.16, <.01) | 2.24 (1.36 - 3.69, <.01) |
| Surgery & radiation | 1.86 (0.78 - 4.45, 0.16) | 2.03 (0.84 - 4.91, 0.12) |
| Radiation alone | 1.75 (1.03 - 2.99, 0.04) | 1.95 (1.10 - 3.44, 0.02) |
| # of RT Fractions |  |  |
| 25 - 29 | Reference | Reference |
| 30 - 34 | 1.03 (0.78 - 1.37, 0.82) | 0.91 (0.68 - 1.21, 0.50) |
| 35 - 40 | 0.98 (0.73 - 1.30, 0.87) | 0.83 (0.62 - 1.13, 0.23) |
| Type of Treatment Center |  |  |
| Free Standing | Reference | Reference |
| Hospital Based | 1.31 (1.05 - 1.64, 0.02) | * |
| Both | 1.61 (0.51 - 5.06, 0.42) | * |
| Rural vs. Urban |  |  |
| Rural | Reference | Reference |
| Urban | 1.07 (0.82 - 1.39, 0.62) | * |
| Radiation Oncologist Density |  |  |
| 1st quartile | Reference | Reference |
| 2nd quartile | 1.09 (0.84 - 1.43, 0.52) | * |
| 3rd quartile | 1.20 (0.91 - 1.58, 0.19) | * |
| 4th quartile | 1.11 (0.82 - 1.50, 0.52) | * |
| Unknown | 1.10 (0.41 - 2.99, 0.85) | * |
| General Surgeon Density |  |  |
| 1st quartile | Reference | Reference |
| 2nd quartile | 1.08 (0.81 - 1.45, 0.59) | * |
| 3rd quartile | 1.35 (1.03 - 1.78, 0.03) | * |
| 4th quartile | 1.35 (1.01 - 1.79, 0.04) | * |
| Unknown | 1.19 (0.44 - 3.24, 0.73) | * |
| Physician Experience |  |  |
| 1st quartile | Reference | Reference |
| 2nd quartile | 1.11 (0.83 - 1.48, 0.48) | * |
| 3rd quartile | 1.07 (0.80 - 1.43, 0.65) | * |
| 4th quartile | 1.07 (0.80 - 1.43, 0.64) | * |
| State |  |  |
| California | Reference | Reference |
| Connecticut | 1.33 (0.88 - 2.02, 0.17) | 1.31 (0.86 - 1.98, 0.21) |
| Georgia | 0.96 (0.67 - 1.38, 0.81) | 0.98 (0.68 - 1.42, 0.92) |
| Hawaii | 1.14 (0.42 - 3.12, 0.80) | 1.17 (0.43 - 3.20, 0.76) |
| Iowa | 0.90 (0.56 - 1.47, 0.68) | 0.89 (0.55 - 1.45, 0.64) |
| Kentucky | 0.72 (0.46 - 1.12, 0.14) | 0.72 (0.46 - 1.13, 0.16) |
| Louisiana | 1.32 (0.88 - 1.98, 0.18) | 1.30 (0.86 - 1.96, 0.21) |
| Michigan | 1.17 (0.77 - 1.78, 0.46) | 1.17 (0.77 - 1.78, 0.46) |
| New Jersey | 1.49 (1.07 - 2.07, 0.02) | 1.49 (1.07 - 2.08, 0.02) |
| New Mexico | 0.74 (0.27 - 2.01, 0.55) | 0.76 (0.28 - 2.07, 0.59) |
| Utah | 1.41 (0.52 - 3.86, 0.50) | 1.48 (0.54 - 4.08, 0.45) |
| Washington | 1.71 (1.15 - 2.56, <.01) | 1.73 (1.15 - 2.59, <.01) |
| Year of Diagnosis |  |  |
| 2006 | Reference | Reference |
| 2007 | 1.04 (0.76 - 1.44, 0.79) | * |
| 2008 | 0.94 (0.67 - 1.31, 0.70) | * |
| 2009 | 1.10 (0.79 - 1.52, 0.57) | * |
| 2010 | 0.60 (0.40 - 0.89, 0.01) | * |
| 2011 | 0.94 (0.67 - 1.33, 0.75) | * |
| IMRT |  |  |
| No | Reference | Reference |
| Yes | 1.11 (0.88 - 1.40, 0.38) | * |
| ^X^ Multivariate Cox regressions were performed using stepwise forward and backwards elimination with threshold values of p ≤ 0.20 and p ≤ 0.05, respectively.  * Covariate auto-excluded from model during forward or backward selection.  Abbrev: HR, hazard ratio. CI, confidence interval. | | |
