# Supplementary material for: Efficacy and cost of high-frequency IGRT in elderly stage III non-small-cell lung cancer patients
Source: PLoS One. 2021 May 27;16(5):e0252053. doi: 10.1371/journal.pone.0252053 (PMC8158910; doi:10.1371/journal.pone.0252053)
Supplement: S8 Table — (DOCX) [file pone.0252053.s013.docx]

|  | | |
| --- | --- | --- |
| Parameter | Univariate  HR (95% CI, P-value) | Multivariate  HR (95% CI, P-Value) |
| Daily IGRT |  |  |
| No | Reference | Reference |
| Yes | 1.12 (1.00 - 1.24, 0.04) | 1.05 (0.93 - 1.18, 0.44) |
| Age |  |  |
| 65 - 74 | Reference | Reference |
| 75 - 84 | 1.03 (0.94 - 1.13, 0.55) | 1.10 (1.00 - 1.21, 0.04) |
| 85+ | 0.65 (0.52 - 0.81, <.01) | 0.83 (0.66 - 1.04, 0.11) |
| Race |  |  |
| White | Reference | Reference |
| Black | 0.85 (0.72 - 1.00, 0.05) | * |
| Hispanic | 1.08 (0.66 - 1.76, 0.76) | * |
| Other | 0.96 (0.77 - 1.21, 0.74) | * |
| COPD |  |  |
| No | Reference | Reference |
| Yes | 1.06 (0.97 - 1.15, 0.23) | * |
| Charlson Score (no COPD) |  |  |
| 0 | Reference | Reference |
| 1-2 | 0.99 (0.90 - 1.09, 0.84) | * |
| > 2 | 1.03 (0.88 - 1.20, 0.71) | * |
| Supplemental O2 |  |  |
| No | Reference | Reference |
| Yes | 0.98 (0.88 - 1.08, 0.66) | 0.98 (0.88 - 1.09, 0.69) |
| Homebound |  |  |
| No | Reference | Reference |
| Yes | 1.13 (0.82 - 1.57, 0.46) | 1.26 (0.90 - 1.75, 0.17) |
| Stage |  |  |
| Stage IIIA | Reference | Reference |
| Stage IIIB | 1.17 (1.07 - 1.27, <.01) | 1.13 (1.03 - 1.24, <.01) |
| T-Stage |  |  |
| TX | Reference | Reference |
| T0 | 0.52 (0.23 - 1.19, 0.12) | * |
| T1 | 0.88 (0.69 - 1.12, 0.30) | * |
| T2 | 0.96 (0.77 - 1.20, 0.71) | * |
| T3 | 0.86 (0.67 - 1.12, 0.26) | * |
| T4 | 0.98 (0.79 - 1.22, 0.86) | * |
| Tumor Size |  |  |
| < 2.0 | Reference | Reference |
| 2.0-5.0 | 1.05 (0.88 - 1.27, 0.58) | * |
| > 5.0 | 1.07 (0.88 - 1.29, 0.52) | * |
| Unknown | 1.24 (1.01 - 1.53, 0.04) | * |
| Histology |  |  |
| Adenocarcinoma | Reference | Reference |
| SCC | 1.07 (0.97 - 1.19, 0.18) | * |
| Large Cell | 1.05 (0.81 - 1.36, 0.73) | * |
| Other | 1.12 (0.99 - 1.27, 0.07) | * |
| Laterality |  |  |
| Right | Reference | Reference |
| Left | 0.96 (0.88 - 1.05, 0.39) | * |
| Unpaired | 0.31 (0.04 - 2.23, 0.25) | * |
| Unknown | 1.17 (0.75 - 1.83, 0.48) | * |
| Tumor Location |  |  |
| Main bronchus | Reference | Reference |
| Upper lobe | 0.73 (0.62 - 0.88, <.01) | 0.77 (0.65 - 0.92, <.01) |
| Middle lobe | 0.57 (0.42 - 0.78, <.01) | 0.62 (0.45 - 0.85, <.01) |
| Lower lobe | 0.88 (0.73 - 1.05, 0.16) | 0.92 (0.76 - 1.11, 0.40) |
| Lung NOS | 0.91 (0.71 - 1.16, 0.44) | 0.88 (0.69 - 1.13, 0.33) |
| Other | 0.71 (0.40 - 1.25, 0.23) | 0.82 (0.46 - 1.45, 0.49) |
| PET |  |  |
| No | Reference | Reference |
| Yes | 1.13 (0.95 - 1.34, 0.17) | * |
| # of Positive Nodes |  |  |
| 0 | Reference | Reference |
| 1-3 | 1.08 (0.84 - 1.40, 0.55) | * |
| 4+ | 1.05 (0.74 - 1.48, 0.78) | * |
| Unknown | 1.20 (0.96 - 1.50, 0.11) | * |
| Treatment Type |  |  |
| Trimodality | Reference | Reference |
| Chemotherapy & radiation | 1.37 (1.14 - 1.63, <.01) | 1.61 (1.33 - 1.94, <.01) |
| Surgery & radiation | 0.53 (0.33 - 0.85, <.01) | 0.53 (0.33 - 0.85, <.01) |
| Radiation alone | 0.76 (0.61 - 0.94, 0.01) | 0.85 (0.68 - 1.07, 0.16) |
| # of RT Fractions |  |  |
| 25 - 29 | Reference | Reference |
| 30 - 34 | 0.74 (0.66 - 0.83, <.01) | 0.68 (0.60 - 0.77, <.01) |
| 35 - 40 | 0.67 (0.59 - 0.75, <.01) | 0.58 (0.51 - 0.66, <.01) |
| Type of Treatment Center |  |  |
| Free Standing | Reference | Reference |
| Hospital Based | 1.00 (0.91 - 1.10, 0.97) | * |
| Both | 1.16 (0.67 - 2.01, 0.59) | * |
| Rural vs. Urban |  |  |
| Rural | Reference | Reference |
| Urban | 0.98 (0.88 - 1.10, 0.72) | * |
| Radiation Oncologist Density |  |  |
| 1st quartile | Reference | Reference |
| 2nd quartile | 0.97 (0.87 - 1.09, 0.60) | 0.80 (0.69 - 0.94, <.01) |
| 3rd quartile | 0.84 (0.74 - 0.95, <.01) | 0.77 (0.64 - 0.92, <.01) |
| 4th quartile | 0.83 (0.73 - 0.95, <.01) | 0.66 (0.54 - 0.80, <.01) |
| Unknown | 0.64 (0.39 - 1.05, 0.08) | 0.55 (0.33 - 0.91, 0.02) |
| General Surgeon Density |  |  |
| 1st quartile | Reference | Reference |
| 2nd quartile | 1.13 (1.00 - 1.27, 0.05) | 1.26 (1.09 - 1.47, <.01) |
| 3rd quartile | 0.93 (0.82 - 1.05, 0.24) | 1.22 (1.01 - 1.47, 0.04) |
| 4th quartile | 1.00 (0.88 - 1.13, 0.97) | 1.32 (1.09 - 1.61, <.01) |
| Unknown | 0.70 (0.43 - 1.16, 0.16) | * |
| Physician Experience |  |  |
| 1st quartile | Reference | Reference |
| 2nd quartile | 0.94 (0.83 - 1.07, 0.33) | * |
| 3rd quartile | 0.95 (0.84 - 1.07, 0.41) | * |
| 4th quartile | 0.86 (0.75 - 0.97, 0.02) | * |
| State |  |  |
| California | Reference | Reference |
| Connecticut | 1.00 (0.83 - 1.21, 0.99) | 1.04 (0.84 - 1.29, 0.71) |
| Georgia | 0.78 (0.67 - 0.90, <.01) | 0.80 (0.68 - 0.94, <.01) |
| Hawaii | 0.60 (0.36 - 0.98, 0.04) | * |
| Iowa | 0.73 (0.59 - 0.89, <.01) | 0.78 (0.63 - 0.96, 0.02) |
| Kentucky | 0.84 (0.71 - 0.99, 0.04) | 0.86 (0.72 - 1.03, 0.10) |
| Louisiana | 0.96 (0.80 - 1.14, 0.61) | 0.86 (0.71 - 1.04, 0.13) |
| Michigan | 0.67 (0.55 - 0.82, <.01) | 0.67 (0.51 - 0.88, <.01) |
| New Jersey | 0.79 (0.68 - 0.92, <.01) | 0.77 (0.64 - 0.91, <.01) |
| New Mexico | 0.86 (0.60 - 1.23, 0.40) | 0.75 (0.52 - 1.08, 0.12) |
| Utah | 0.73 (0.44 - 1.23, 0.24) | 0.64 (0.38 - 1.07, 0.09) |
| Washington | 0.94 (0.77 - 1.15, 0.56) | 0.96 (0.76 - 1.20, 0.70) |
| Year of Diagnosis |  |  |
| 2006 | Reference | Reference |
| 2007 | 1.01 (0.87 - 1.16, 0.94) | * |
| 2008 | 0.89 (0.76 - 1.03, 0.12) | * |
| 2009 | 1.03 (0.88 - 1.19, 0.74) | * |
| 2010 | 0.99 (0.85 - 1.16, 0.93) | * |
| 2011 | 1.02 (0.87 - 1.19, 0.83) | * |
| IMRT |  |  |
| No | Reference | Reference |
| Yes | 1.22 (1.10 - 1.35, <.01) | 1.20 (1.06 - 1.34, <.01) |
| ^X^ Multivariate Cox regressions were performed using stepwise forward and backwards elimination with threshold values of p ≤ 0.20 and p ≤ 0.05, respectively.  * Covariate auto-excluded from model during forward or backward selection.  Abbrev: HR, hazard ratio. CI, confidence interval. | | |
