# Supplementary material for: Efficacy and cost of high-frequency IGRT in elderly stage III non-small-cell lung cancer patients
Source: PLoS One. 2021 May 27;16(5):e0252053. doi: 10.1371/journal.pone.0252053 (PMC8158910; doi:10.1371/journal.pone.0252053)
Supplement: S10 Table — (DOCX) [file pone.0252053.s015.docx]

|  | | |
| --- | --- | --- |
| Parameter | Univariate  HR (95% CI, P-value) | Multivariate  HR (95% CI, P-Value) |
| Daily IGRT |  |  |
| No | Reference | Reference |
| Yes | 1.13 (1.00 - 1.28, 0.04) | 1.06 (0.92 - 1.21, 0.41) |
| Age |  |  |
| 65 - 74 | Reference | Reference |
| 75 - 84 | 1.10 (0.99 - 1.22, 0.07) | 1.19 (1.07 - 1.32, <.01) |
| 85+ | 0.68 (0.53 - 0.88, <.01) | 0.92 (0.71 - 1.19, 0.52) |
| Race |  |  |
| White | Reference | Reference |
| Black | 0.72 (0.59 - 0.87, <.01) | 0.76 (0.62 - 0.93, <.01) |
| Hispanic | 0.78 (0.42 - 1.46, 0.44) | 0.74 (0.40 - 1.38, 0.34) |
| Other | 1.00 (0.78 - 1.29, 0.98) | 0.94 (0.72 - 1.24, 0.67) |
| COPD |  |  |
| No | Reference | Reference |
| Yes | 1.02 (0.93 - 1.13, 0.64) | * |
| Charlson Score (no COPD) |  |  |
| 0 | Reference | Reference |
| 1-2 | 0.96 (0.86 - 1.08, 0.51) | * |
| > 2 | 0.90 (0.75 - 1.08, 0.28) | * |
| Supplemental O2 |  |  |
| No | Reference | Reference |
| Yes | 0.98 (0.87 - 1.10, 0.73) | 0.99 (0.88 - 1.11, 0.85) |
| Homebound |  |  |
| No | Reference | Reference |
| Yes | 1.10 (0.76 - 1.60, 0.62) | 1.24 (0.85 - 1.82, 0.27) |
| Stage |  |  |
| Stage IIIA | Reference | Reference |
| Stage IIIB | 1.18 (1.07 - 1.31, <.01) | 1.13 (1.02 - 1.25, 0.02) |
| T-Stage |  |  |
| TX | Reference | Reference |
| T0 | 0.39 (0.14 - 1.06, 0.07) | * |
| T1 | 0.75 (0.58 - 0.98, 0.04) | * |
| T2 | 0.88 (0.69 - 1.12, 0.29) | * |
| T3 | 0.77 (0.58 - 1.02, 0.06) | * |
| T4 | 0.90 (0.70 - 1.14, 0.37) | * |
| Tumor Size |  |  |
| < 2.0 | Reference | Reference |
| 2.0-5.0 | 1.13 (0.91 - 1.40, 0.26) | * |
| > 5.0 | 1.15 (0.92 - 1.44, 0.21) | * |
| Unknown | 1.32 (1.04 - 1.67, 0.02) | * |
| Histology |  |  |
| Adenocarcinoma | Reference | Reference |
| SCC | 1.05 (0.93 - 1.18, 0.43) | * |
| Large Cell | 1.03 (0.76 - 1.39, 0.84) | * |
| Other | 1.14 (0.99 - 1.31, 0.07) | * |
| Laterality |  |  |
| Right | Reference | Reference |
| Left | 0.92 (0.83 - 1.02, 0.12) | * |
| Unpaired | 0.00 (0.00 - 4E78, 0.92) | * |
| Unknown | 0.84 (0.49 - 1.46, 0.54) | * |
| Tumor Location |  |  |
| Main bronchus | Reference | Reference |
| Upper lobe | 0.76 (0.62 - 0.94, <.01) | 0.81 (0.66 - 1.00, 0.05) |
| Middle lobe | 0.64 (0.45 - 0.91, 0.01) | 0.72 (0.50 - 1.02, 0.07) |
| Lower lobe | 1.00 (0.81 - 1.24, 0.99) | 1.06 (0.85 - 1.31, 0.61) |
| Lung NOS | 1.02 (0.77 - 1.34, 0.92) | 0.99 (0.75 - 1.32, 0.97) |
| Other | 0.94 (0.52 - 1.71, 0.84) | 1.13 (0.62 - 2.05, 0.70) |
| PET |  |  |
| No | Reference | Reference |
| Yes | 1.12 (0.92 - 1.36, 0.26) | * |
| # of Positive Nodes |  |  |
| 0 | Reference | Reference |
| 1-3 | 0.99 (0.74 - 1.33, 0.97) | * |
| 4+ | 1.06 (0.71 - 1.56, 0.78) | * |
| Unknown | 1.21 (0.94 - 1.57, 0.14) | * |
| Treatment Type |  |  |
| Trimodality | Reference | Reference |
| Chemotherapy & radiation | 1.51 (1.23 - 1.86, <.01) | 1.80 (1.44 - 2.24, <.01) |
| Surgery & radiation | 0.55 (0.31 - 0.96, 0.04) | 0.53 (0.30 - 0.94, 0.03) |
| Radiation alone | 0.72 (0.55 - 0.93, 0.01) | 0.79 (0.60 - 1.05, 0.10) |
| # of RT Fractions |  |  |
| 25 - 29 | Reference | Reference |
| 30 - 34 | 0.75 (0.66 - 0.85, <.01) | 0.67 (0.59 - 0.77, <.01) |
| 35 - 40 | 0.65 (0.56 - 0.74, <.01) | 0.55 (0.48 - 0.64, <.01) |
| Type of Treatment Center |  |  |
| Free Standing | Reference | Reference |
| Hospital Based | 0.96 (0.87 - 1.07, 0.48) | * |
| Both | 1.21 (0.67 - 2.20, 0.53) | * |
| Rural vs. Urban |  |  |
| Rural | Reference | Reference |
| Urban | 1.06 (0.93 - 1.21, 0.34) | * |
| Radiation Oncologist Density |  |  |
| 1st quartile | Reference | Reference |
| 2nd quartile | 1.05 (0.92 - 1.19, 0.50) | 0.86 (0.72 - 1.03, 0.09) |
| 3rd quartile | 0.91 (0.79 - 1.05, 0.18) | 0.80 (0.66 - 0.98, 0.03) |
| 4th quartile | 0.84 (0.72 - 0.98, 0.03) | 0.66 (0.53 - 0.83, <.01) |
| Unknown | 0.74 (0.43 - 1.29, 0.29) | 0.63 (0.35 - 1.13, 0.12) |
| General Surgeon Density |  |  |
| 1st quartile | Reference | Reference |
| 2nd quartile | 1.17 (1.02 - 1.34, 0.03) | 1.27 (1.07 - 1.51, <.01) |
| 3rd quartile | 0.99 (0.86 - 1.13, 0.83) | 1.22 (0.99 - 1.52, 0.06) |
| 4th quartile | 1.00 (0.87 - 1.16, 0.96) | 1.33 (1.06 - 1.66, 0.01) |
| Unknown | 0.80 (0.46 - 1.39, 0.43) | * |
| Physician Experience |  |  |
| 1st quartile | Reference | Reference |
| 2nd quartile | 0.93 (0.81 - 1.07, 0.31) | * |
| 3rd quartile | 0.88 (0.77 - 1.02, 0.08) | * |
| 4th quartile | 0.80 (0.69 - 0.92, <.01) | * |
| State |  |  |
| California | Reference | Reference |
| Connecticut | 1.07 (0.87 - 1.31, 0.53) | 1.09 (0.86 - 1.38, 0.47) |
| Georgia | 0.74 (0.62 - 0.87, <.01) | 0.77 (0.65 - 0.93, <.01) |
| Hawaii | 0.65 (0.37 - 1.12, 0.12) | * |
| Iowa | 0.70 (0.56 - 0.88, <.01) | 0.75 (0.59 - 0.95, 0.02) |
| Kentucky | 0.78 (0.65 - 0.94, <.01) | 0.81 (0.66 - 1.00, 0.04) |
| Louisiana | 0.83 (0.68 - 1.02, 0.07) | 0.78 (0.63 - 0.98, 0.03) |
| Michigan | 0.71 (0.57 - 0.88, <.01) | 0.71 (0.52 - 0.97, 0.03) |
| New Jersey | 0.72 (0.61 - 0.86, <.01) | 0.68 (0.56 - 0.83, <.01) |
| New Mexico | 0.88 (0.59 - 1.31, 0.52) | 0.79 (0.53 - 1.19, 0.27) |
| Utah | 0.84 (0.48 - 1.46, 0.54) | 0.72 (0.41 - 1.27, 0.26) |
| Washington | 1.01 (0.81 - 1.25, 0.94) | 1.01 (0.78 - 1.30, 0.93) |
| Year of Diagnosis |  |  |
| 2006 | Reference | Reference |
| 2007 | 1.07 (0.90 - 1.26, 0.45) | * |
| 2008 | 0.91 (0.77 - 1.09, 0.31) | * |
| 2009 | 1.09 (0.92 - 1.30, 0.29) | * |
| 2010 | 1.03 (0.87 - 1.23, 0.71) | * |
| 2011 | 1.05 (0.88 - 1.26, 0.57) | * |
| IMRT |  |  |
| No | Reference | Reference |
| Yes | 1.22 (1.08 - 1.37, <.01) | 1.18 (1.03 - 1.35, 0.01) |
| ^X^ Multivariate Cox regressions were performed using stepwise forward and backwards elimination with threshold values of p ≤ 0.20 and p ≤ 0.05, respectively.  * Covariate auto-excluded from model during forward or backward selection.  Abbrev: HR, hazard ratio. CI, confidence interval. | | |
