# Supplementary material for: Efficacy and cost of high-frequency IGRT in elderly stage III non-small-cell lung cancer patients
Source: PLoS One. 2021 May 27;16(5):e0252053. doi: 10.1371/journal.pone.0252053 (PMC8158910; doi:10.1371/journal.pone.0252053)
Supplement: S11 Table — (DOCX) [file pone.0252053.s016.docx]

|  | | |
| --- | --- | --- |
| Parameter | Univariate  HR (95% CI, P-value) | Multivariate  HR (95% CI, P-Value) |
| Daily IGRT |  |  |
| No | Reference | Reference |
| Yes | 1.16 (0.81 - 1.65, 0.43) | 1.07 (0.74 - 1.54, 0.72) |
| Age |  |  |
| 65 - 74 | Reference | Reference |
| 75 - 84 | 0.86 (0.64 - 1.16, 0.34) | 0.91 (0.67 - 1.24, 0.54) |
| 85+ | 0.36 (0.11 - 1.13, 0.08) | 0.46 (0.14 - 1.49, 0.19) |
| Race |  |  |
| White | Reference | Reference |
| Black | 0.91 (0.56 - 1.50, 0.72) | * |
| Hispanic | 0.99 (0.14 - 7.06, 0.99) | * |
| Other | 1.10 (0.58 - 2.09, 0.77) | * |
| COPD |  |  |
| No | Reference | Reference |
| Yes | 1.18 (0.89 - 1.56, 0.26) | * |
| Charlson Score (no COPD) |  |  |
| 0 | Reference | Reference |
| 1-2 | 0.83 (0.61 - 1.12, 0.22) | * |
| > 2 | 0.69 (0.38 - 1.24, 0.22) | * |
| Supplemental O2 |  |  |
| No | Reference | Reference |
| Yes | 1.02 (0.72 - 1.46, 0.89) | 0.97 (0.67 - 1.40, 0.87) |
| Homebound |  |  |
| No | Reference | Reference |
| Yes | 0.47 (0.12 - 1.90, 0.29) | 0.29 (0.07 - 1.26, 0.10) |
| Stage |  |  |
| Stage IIIA | Reference | Reference |
| Stage IIIB | 1.46 (1.10 - 1.95, <.01) | 1.44 (1.07 - 1.94, 0.02) |
| T-Stage |  |  |
| TX | Reference | Reference |
| T0 | 1.62 (0.44 - 6.02, 0.47) | * |
| T1 | 1.35 (0.64 - 2.86, 0.43) | * |
| T2 | 1.07 (0.52 - 2.18, 0.86) | * |
| T3 | 0.98 (0.44 - 2.17, 0.95) | * |
| T4 | 1.49 (0.75 - 2.98, 0.26) | * |
| Tumor Size |  |  |
| < 2.0 | Reference | Reference |
| 2.0-5.0 | 1.17 (0.63 - 2.18, 0.62) | * |
| > 5.0 | 0.95 (0.49 - 1.82, 0.87) | * |
| Unknown | 1.19 (0.60 - 2.36, 0.61) | * |
| Histology |  |  |
| Adenocarcinoma | Reference | Reference |
| SCC | 1.31 (0.94 - 1.84, 0.11) | * |
| Large Cell | 1.89 (0.81 - 4.43, 0.14) | * |
| Other | 1.22 (0.81 - 1.85, 0.34) | * |
| Laterality |  |  |
| Right | Reference | Reference |
| Left | 1.05 (0.79 - 1.40, 0.74) | * |
| Unpaired | - | * |
| Unknown | 0.67 (0.16 - 2.70, 0.57) | * |
| Tumor Location |  |  |
| Main bronchus | Reference | Reference |
| Upper lobe | 0.51 (0.29 - 0.88, 0.02) | 0.60 (0.34 - 1.06, 0.08) |
| Middle lobe | 0.53 (0.21 - 1.37, 0.19) | 0.77 (0.29 - 2.01, 0.59) |
| Lower lobe | 0.81 (0.46 - 1.43, 0.46) | 1.02 (0.57 - 1.83, 0.94) |
| Lung NOS | 0.55 (0.27 - 1.13, 0.10) | 0.61 (0.29 - 1.28, 0.19) |
| Other | 0.66 (0.09 - 5.00, 0.69) | 0.77 (0.10 - 5.92, 0.81) |
| PET |  |  |
| No | Reference | Reference |
| Yes | 1.14 (0.66 - 1.97, 0.63) | * |
| # of Positive Nodes |  |  |
| 0 | Reference | Reference |
| 1-3 | 1.08 (0.49 - 2.40, 0.85) | * |
| 4+ | 0.95 (0.34 - 2.64, 0.93) | * |
| Unknown | 1.11 (0.54 - 2.26, 0.77) | * |
| Treatment Type |  |  |
| Trimodality | Reference | Reference |
| Chemotherapy & radiation | 1.05 (0.65 - 1.69, 0.85) | 1.19 (0.70 - 2.01, 0.52) |
| Surgery & radiation | 0.35 (0.05 - 2.62, 0.31) | 0.38 (0.05 - 2.86, 0.35) |
| Radiation alone | 0.64 (0.34 - 1.23, 0.18) | 0.77 (0.38 - 1.58, 0.48) |
| # of RT Fractions |  |  |
| 25 - 29 | Reference | Reference |
| 30 - 34 | 0.63 (0.45 - 0.89, <.01) | 0.55 (0.38 - 0.79, <.01) |
| 35 - 40 | 0.50 (0.34 - 0.72, <.01) | 0.42 (0.29 - 0.63, <.01) |
| Type of Treatment Center |  |  |
| Free Standing | Reference | Reference |
| Hospital Based | 0.88 (0.66 - 1.19, 0.41) | * |
| Both | 1.19 (0.16 - 8.56, 0.87) | * |
| Rural vs. Urban |  |  |
| Rural | Reference | Reference |
| Urban | 0.76 (0.54 - 1.08, 0.13) | * |
| Radiation Oncologist Density |  |  |
| 1st quartile | Reference | Reference |
| 2nd quartile | 0.47 (0.32 - 0.69, <.01) | 0.47 (0.32 - 0.69, <.01) |
| 3rd quartile | 0.80 (0.56 - 1.15, 0.23) | 0.86 (0.60 - 1.25, 0.44) |
| 4th quartile | 0.43 (0.27 - 0.69, <.01) | 0.38 (0.24 - 0.61, <.01) |
| Unknown | 0.53 (0.07 - 3.83, 0.53) | 0.48 (0.07 - 3.44, 0.46) |
| General Surgeon Density |  |  |
| 1st quartile | Reference | Reference |
| 2nd quartile | 0.81 (0.57 - 1.16, 0.25) | * |
| 3rd quartile | 0.53 (0.36 - 0.78, <.01) | * |
| 4th quartile | 0.46 (0.30 - 0.72, <.01) | * |
| Unknown | 0.54 (0.08 - 3.91, 0.54) | * |
| Physician Experience |  |  |
| 1st quartile | Reference | Reference |
| 2nd quartile | 1.12 (0.75 - 1.69, 0.58) | * |
| 3rd quartile | 1.02 (0.69 - 1.51, 0.92) | * |
| 4th quartile | 1.25 (0.84 - 1.84, 0.27) | * |
| State |  |  |
| California | Reference | Reference |
| Connecticut | 0.52 (0.24 - 1.11, 0.09) | * |
| Georgia | 1.14 (0.72 - 1.83, 0.57) | * |
| Hawaii | 0.78 (0.11 - 5.70, 0.81) | * |
| Iowa | 0.78 (0.39 - 1.57, 0.49) | * |
| Kentucky | 1.39 (0.86 - 2.23, 0.17) | * |
| Louisiana | 1.73 (1.05 - 2.85, 0.03) | * |
| Michigan | 0.94 (0.52 - 1.70, 0.84) | * |
| New Jersey | 0.95 (0.57 - 1.56, 0.83) | * |
| New Mexico | 1.37 (0.49 - 3.84, 0.55) | * |
| Utah | 1.98 (0.48 - 8.21, 0.35) | * |
| Washington | 0.53 (0.21 - 1.35, 0.18) | * |
| Year of Diagnosis |  |  |
| 2006 | Reference | Reference |
| 2007 | 1.13 (0.72 - 1.76, 0.60) | * |
| 2008 | 0.96 (0.60 - 1.53, 0.85) | * |
| 2009 | 1.14 (0.71 - 1.84, 0.59) | * |
| 2010 | 1.07 (0.64 - 1.79, 0.80) | * |
| 2011 | 1.30 (0.78 - 2.16, 0.32) | * |
| IMRT |  |  |
| No | Reference | Reference |
| Yes | 1.08 (0.78 - 1.49, 0.65) | * |
| ^X^ Multivariate Cox regressions were performed using stepwise forward and backwards elimination with threshold values of p ≤ 0.20 and p ≤ 0.05, respectively.  * Covariate auto-excluded from model during forward or backward selection.  Abbrev: HR, hazard ratio. CI, confidence interval. | | |
