# Supplementary material for: Efficacy and cost of high-frequency IGRT in elderly stage III non-small-cell lung cancer patients
Source: PLoS One. 2021 May 27;16(5):e0252053. doi: 10.1371/journal.pone.0252053 (PMC8158910; doi:10.1371/journal.pone.0252053)
Supplement: S12 Table — (DOCX) [file pone.0252053.s017.docx]

|  | | |
| --- | --- | --- |
| Parameter | Univariate  HR (95% CI, P-value) | Multivariate  HR (95% CI, P-Value) |
| Daily IGRT |  |  |
| No | Reference | Reference |
| Yes | 1.70 (1.03 - 2.78, 0.04) | 1.70 (1.03 - 2.80, 0.04) |
| Age |  |  |
| 65 - 74 | Reference | Reference |
| 75 - 84 | 0.72 (0.44 - 1.19, 0.20) | 0.93 (0.56 - 1.54, 0.77) |
| 85+ | 0.84 (0.30 - 2.34, 0.74) | 1.93 (0.68 - 5.44, 0.22) |
| Race |  |  |
| White | Reference | Reference |
| Black | 0.77 (0.31 - 1.92, 0.58) | * |
| Hispanic | 1.73 (0.24 - 12.5, 0.59) | * |
| Other | 0.65 (0.16 - 2.67, 0.55) | * |
| COPD |  |  |
| No | Reference | Reference |
| Yes | 1.24 (0.78 - 1.97, 0.36) | * |
| Charlson Score (no COPD) |  |  |
| 0 | Reference | Reference |
| 1-2 | 1.10 (0.68 - 1.80, 0.70) | * |
| > 2 | 0.94 (0.40 - 2.21, 0.88) | * |
| Supplemental O2 |  |  |
| No | Reference | Reference |
| Yes | 1.36 (0.82 - 2.25, 0.23) | 1.45 (0.87 - 2.40, 0.15) |
| Homebound |  |  |
| No | Reference | Reference |
| Yes | - | 0.00 (0.00 - . , 0.98) |
| Stage |  |  |
| Stage IIIA | Reference | Reference |
| Stage IIIB | 1.40 (0.88 - 2.24, 0.16) | 1.41 (0.87 - 2.26, 0.16) |
| T-Stage |  |  |
| TX | Reference | Reference |
| T0 | - | * |
| T1 | 0.84 (0.22 - 3.18, 0.80) | * |
| T2 | 1.01 (0.30 - 3.38, 0.98) | * |
| T3 | 1.23 (0.33 - 4.65, 0.76) | * |
| T4 | 1.15 (0.35 - 3.75, 0.82) | * |
| Tumor Size |  |  |
| < 2.0 | Reference | Reference |
| 2.0-5.0 | 0.65 (0.29 - 1.49, 0.31) | * |
| > 5.0 | 0.78 (0.34 - 1.81, 0.56) | * |
| Unknown | 0.67 (0.26 - 1.77, 0.42) | * |
| Histology |  |  |
| Adenocarcinoma | Reference | Reference |
| SCC | 1.04 (0.61 - 1.76, 0.90) | * |
| Large Cell | 1.37 (0.41 - 4.54, 0.61) | * |
| Other | 0.90 (0.46 - 1.73, 0.74) | * |
| Laterality |  |  |
| Right | Reference | Reference |
| Left | 1.15 (0.72 - 1.83, 0.56) | * |
| Unpaired | - | * |
| Unknown | - | * |
| Tumor Location |  |  |
| Main bronchus | Reference | Reference |
| Upper lobe | 0.88 (0.35 - 2.21, 0.78) | * |
| Middle lobe | 0.32 (0.04 - 2.74, 0.30) | * |
| Lower lobe | 1.01 (0.38 - 2.69, 0.98) | * |
| Lung NOS | 0.23 (0.03 - 1.93, 0.17) | * |
| Other | 1.65 (0.19 - 14.1, 0.65) | * |
| PET |  |  |
| No | Reference | Reference |
| Yes | 1.83 (0.58 - 5.81, 0.31) | * |
| # of Positive Nodes |  |  |
| 0 | Reference | Reference |
| 1-3 | 1.11 (0.30 - 4.10, 0.87) | * |
| 4+ | 1.54 (0.31 - 7.63, 0.60) | * |
| Unknown | 1.14 (0.36 - 3.63, 0.83) | * |
| Treatment Type |  |  |
| Trimodality | Reference | Reference |
| Chemotherapy & radiation | 1.03 (0.47 - 2.25, 0.94) | 1.08 (0.47 - 2.47, 0.86) |
| Surgery & radiation | - | 0.00 (0.00 - . , 0.98) |
| Radiation alone | 0.08 (0.01 - 0.61, 0.02) | 0.07 (0.01 - 0.58, 0.01) |
| # of RT Fractions |  |  |
| 25 - 29 | Reference | Reference |
| 30 - 34 | 0.64 (0.36 - 1.13, 0.12) | 0.64 (0.36 - 1.15, 0.14) |
| 35 - 40 | 0.58 (0.32 - 1.06, 0.08) | 0.52 (0.28 - 0.98, 0.04) |
| Type of Treatment Center |  |  |
| Free Standing | Reference | Reference |
| Hospital Based | 1.09 (0.67 - 1.79, 0.73) | * |
| Both | - | * |
| Rural vs. Urban |  |  |
| Rural | Reference | Reference |
| Urban | 0.49 (0.30 - 0.81, <.01) | 0.49 (0.30 - 0.81, <.01) |
| Radiation Oncologist Density |  |  |
| 1st quartile | Reference | Reference |
| 2nd quartile | 0.79 (0.45 - 1.39, 0.41) | * |
| 3rd quartile | 0.69 (0.37 - 1.29, 0.25) | * |
| 4th quartile | 0.43 (0.19 - 0.97, 0.04) | * |
| Unknown | - | * |
| General Surgeon Density |  |  |
| 1st quartile | Reference | Reference |
| 2nd quartile | 0.93 (0.52 - 1.65, 0.81) | * |
| 3rd quartile | 0.58 (0.30 - 1.11, 0.10) | * |
| 4th quartile | 0.57 (0.29 - 1.15, 0.12) | * |
| Unknown | - | * |
| Physician Experience |  |  |
| 1st quartile | Reference | Reference |
| 2nd quartile | 0.84 (0.43 - 1.64, 0.61) | * |
| 3rd quartile | 1.09 (0.59 - 2.04, 0.77) | * |
| 4th quartile | 0.84 (0.43 - 1.64, 0.61) | * |
| State |  |  |
| California | Reference | Reference |
| Connecticut | 0.27 (0.06 - 1.13, 0.07) | * |
| Georgia | 0.64 (0.30 - 1.35, 0.24) | * |
| Hawaii | - | * |
| Iowa | 0.97 (0.42 - 2.25, 0.94) | * |
| Kentucky | 0.63 (0.27 - 1.46, 0.28) | * |
| Louisiana | 1.08 (0.50 - 2.34, 0.84) | * |
| Michigan | 0.24 (0.06 - 1.02, 0.05) | * |
| New Jersey | 0.42 (0.17 - 1.02, 0.06) | * |
| New Mexico | 1.18 (0.28 - 4.99, 0.83) | * |
| Utah | - | * |
| Washington | 0.62 (0.21 - 1.79, 0.38) | * |
| Year of Diagnosis |  |  |
| 2006 | Reference | Reference |
| 2007 | 1.76 (0.78 - 3.98, 0.17) | * |
| 2008 | 1.16 (0.47 - 2.85, 0.75) | * |
| 2009 | 1.81 (0.79 - 4.13, 0.16) | * |
| 2010 | 1.23 (0.49 - 3.10, 0.66) | * |
| 2011 | 1.71 (0.73 - 4.01, 0.21) | * |
| IMRT |  |  |
| No | Reference | Reference |
| Yes | 0.96 (0.55 - 1.68, 0.89) | * |
| ^X^ Multivariate Cox regressions were performed using stepwise forward and backwards elimination with threshold values of p ≤ 0.20 and p ≤ 0.05, respectively.  * Covariate auto-excluded from model during forward or backward selection.  Abbrev: HR, hazard ratio. CI, confidence interval. | | |
