# Supplementary material for: Efficacy and cost of high-frequency IGRT in elderly stage III non-small-cell lung cancer patients
Source: PLoS One. 2021 May 27;16(5):e0252053. doi: 10.1371/journal.pone.0252053 (PMC8158910; doi:10.1371/journal.pone.0252053)
Supplement: S13 Table — (DOCX) [file pone.0252053.s018.docx]

|  | | |
| --- | --- | --- |
| Parameter | Univariate  HR (95% CI, P-value) | Multivariate  HR (95% CI, P-Value) |
| Daily IGRT |  |  |
| No | Reference | Reference |
| Yes | 0.89 (0.81 - 0.97, 0.01) | 0.94 (0.84 - 1.04, 0.24) |
| Age |  |  |
| 65 - 74 | Reference | Reference |
| 75 - 84 | 1.21 (1.12 - 1.30, <.01) | 1.15 (1.06 - 1.24, <.01) |
| 85+ | 1.52 (1.31 - 1.77, <.01) | 1.26 (1.07 - 1.48, <.01) |
| Race |  |  |
| White | Reference | Reference |
| Black | 0.87 (0.76 - 0.99, 0.04) | * |
| Hispanic | 0.88 (0.58 - 1.34, 0.56) | * |
| Other | 0.92 (0.77 - 1.11, 0.38) | * |
| COPD |  |  |
| No | Reference | Reference |
| Yes | 1.16 (1.08 - 1.25, <.01) | 1.12 (1.03 - 1.21, <.01) |
| Charlson Score (no COPD) |  |  |
| 0 | Reference | Reference |
| 1-2 | 1.03 (0.95 - 1.11, 0.52) | 1.00 (0.93 - 1.09, 0.91) |
| > 2 | 1.27 (1.12 - 1.44, <.01) | 1.19 (1.05 - 1.36, <.01) |
| Supplemental O2 |  |  |
| No | Reference | Reference |
| Yes | 1.23 (1.13 - 1.33, <.01) | 1.10 (1.00 - 1.20, 0.05) |
| Homebound |  |  |
| No | Reference | Reference |
| Yes | 1.19 (0.89 - 1.59, 0.24) | 0.88 (0.65 - 1.18, 0.39) |
| Stage |  |  |
| Stage IIIA | Reference | Reference |
| Stage IIIB | 1.27 (1.18 - 1.36, <.01) | 1.11 (0.98 - 1.25, 0.09) |
| T-Stage |  |  |
| TX | Reference | Reference |
| T0 | 0.37 (0.18 - 0.76, <.01) | 0.43 (0.21 - 0.90, 0.02) |
| T1 | 0.75 (0.62 - 0.91, <.01) | 0.97 (0.77 - 1.21, 0.77) |
| T2 | 0.92 (0.77 - 1.10, 0.34) | 1.04 (0.85 - 1.27, 0.72) |
| T3 | 1.08 (0.88 - 1.32, 0.48) | 1.19 (0.96 - 1.48, 0.12) |
| T4 | 1.14 (0.95 - 1.36, 0.15) | 1.12 (0.90 - 1.39, 0.30) |
| Tumor Size |  |  |
| < 2.0 | Reference | Reference |
| 2.0-5.0 | 1.30 (1.11 - 1.53, <.01) | 1.14 (0.97 - 1.36, 0.12) |
| > 5.0 | 1.62 (1.37 - 1.90, <.01) | 1.40 (1.17 - 1.68, <.01) |
| Unknown | 1.68 (1.41 - 1.99, <.01) | 1.44 (1.18 - 1.76, <.01) |
| Histology |  |  |
| Adenocarcinoma | Reference | Reference |
| SCC | 1.23 (1.13 - 1.34, <.01) | * |
| Large Cell | 1.21 (0.98 - 1.49, 0.08) | * |
| Other | 1.22 (1.11 - 1.35, <.01) | * |
| Laterality |  |  |
| Right | Reference | Reference |
| Left | 0.93 (0.86 - 1.00, 0.05) | 0.91 (0.84 - 0.98, 0.01) |
| Unpaired | 0.93 (0.35 - 2.47, 0.88) | 0.70 (0.26 - 1.89, 0.48) |
| Unknown | 0.60 (0.40 - 0.92, 0.02) | 0.66 (0.42 - 1.05, 0.08) |
| Tumor Location |  |  |
| Main bronchus | Reference | Reference |
| Upper lobe | 0.74 (0.64 - 0.86, <.01) | 0.86 (0.74 - 1.01, 0.06) |
| Middle lobe | 0.89 (0.71 - 1.12, 0.32) | 1.05 (0.83 - 1.32, 0.71) |
| Lower lobe | 0.94 (0.80 - 1.10, 0.41) | 1.06 (0.91 - 1.25, 0.45) |
| Lung NOS | 0.76 (0.62 - 0.94, 0.01) | 0.87 (0.69 - 1.08, 0.21) |
| Other | 1.15 (0.76 - 1.73, 0.51) | 1.33 (0.88 - 2.00, 0.18) |
| PET |  |  |
| No | Reference | Reference |
| Yes | 0.85 (0.74 - 0.97, 0.02) | * |
| # of Positive Nodes |  |  |
| 0 | Reference | Reference |
| 1-3 | 0.99 (0.80 - 1.21, 0.89) | 1.19 (0.96 - 1.47, 0.11) |
| 4+ | 1.06 (0.81 - 1.40, 0.66) | 1.31 (0.99 - 1.74, 0.06) |
| Unknown | 1.56 (1.30 - 1.87, <.01) | 1.43 (1.18 - 1.73, <.01) |
| Treatment Type |  |  |
| Trimodality | Reference | Reference |
| Chemotherapy & radiation | 1.80 (1.54 - 2.09, <.01) | 1.59 (1.32 - 1.91, <.01) |
| Surgery & radiation | 1.44 (1.06 - 1.97, 0.02) | 1.36 (0.99 - 1.86, 0.06) |
| Radiation alone | 2.73 (2.30 - 3.24, <.01) | 2.16 (1.76 - 2.65, <.01) |
| # of RT Fractions |  |  |
| 25 - 29 | Reference | Reference |
| 30 - 34 | 0.92 (0.84 - 1.02, 0.10) | 0.77 (0.70 - 0.85, <.01) |
| 35 - 40 | 0.92 (0.83 - 1.01, 0.09) | 0.76 (0.68 - 0.85, <.01) |
| Type of Treatment Center |  |  |
| Free Standing | Reference | Reference |
| Hospital Based | 0.94 (0.87 - 1.01, 0.11) | * |
| Both | 1.08 (0.69 - 1.70, 0.74) | * |
| Rural vs. Urban |  |  |
| Rural | Reference | Reference |
| Urban | 0.92 (0.84 - 1.00, 0.06) | 0.91 (0.83 - 0.99, 0.04) |
| Radiation Oncologist Density |  |  |
| 1st quartile | Reference | Reference |
| 2nd quartile | 0.99 (0.90 - 1.08, 0.76) | * |
| 3rd quartile | 0.92 (0.83 - 1.02, 0.10) | * |
| 4th quartile | 0.91 (0.81 - 1.01, 0.08) | * |
| Unknown | 0.89 (0.62 - 1.28, 0.54) | * |
| General Surgeon Density |  |  |
| 1st quartile | Reference | Reference |
| 2nd quartile | 1.04 (0.94 - 1.14, 0.47) | * |
| 3rd quartile | 0.98 (0.89 - 1.08, 0.72) | * |
| 4th quartile | 0.96 (0.87 - 1.06, 0.43) | * |
| Unknown | 0.92 (0.64 - 1.33, 0.67) | * |
| Physician Experience |  |  |
| 1st quartile | Reference | Reference |
| 2nd quartile | 1.03 (0.93 - 1.14, 0.53) | * |
| 3rd quartile | 0.99 (0.90 - 1.10, 0.89) | * |
| 4th quartile | 1.06 (0.96 - 1.17, 0.28) | * |
| State |  |  |
| California | Reference | Reference |
| Connecticut | 0.87 (0.74 - 1.03, 0.10) | * |
| Georgia | 1.04 (0.92 - 1.18, 0.52) | * |
| Hawaii | 0.96 (0.66 - 1.38, 0.81) | * |
| Iowa | 1.13 (0.97 - 1.32, 0.13) | * |
| Kentucky | 1.04 (0.91 - 1.19, 0.58) | * |
| Louisiana | 1.20 (1.04 - 1.39, 0.01) | * |
| Michigan | 0.95 (0.81 - 1.10, 0.47) | * |
| New Jersey | 1.11 (0.98 - 1.26, 0.09) | * |
| New Mexico | 0.94 (0.69 - 1.27, 0.67) | * |
| Utah | 0.93 (0.61 - 1.40, 0.72) | * |
| Washington | 0.95 (0.80 - 1.12, 0.55) | * |
| Year of Diagnosis |  |  |
| 2006 | Reference | Reference |
| 2007 | 0.89 (0.80 - 1.00, 0.04) | 0.92 (0.82 - 1.03, 0.15) |
| 2008 | 0.82 (0.73 - 0.92, <.01) | 0.81 (0.72 - 0.91, <.01) |
| 2009 | 0.92 (0.82 - 1.04, 0.18) | 0.94 (0.83 - 1.06, 0.34) |
| 2010 | 0.81 (0.71 - 0.92, <.01) | 0.81 (0.71 - 0.93, <.01) |
| 2011 | 0.61 (0.53 - 0.70, <.01) | 0.61 (0.53 - 0.71, <.01) |
| IMRT |  |  |
| No | Reference | Reference |
| Yes | 1.02 (0.94 - 1.11, 0.65) | 1.13 (1.03 - 1.24, 0.01) |
| ^X^ Multivariate Cox regressions were performed using stepwise forward and backwards elimination with threshold values of p ≤ 0.20 and p ≤ 0.05, respectively.  * Covariate auto-excluded from model during forward or backward selection.  Abbrev: HR, hazard ratio. CI, confidence interval. | | |
