# Supplementary material for: Efficacy and cost of high-frequency IGRT in elderly stage III non-small-cell lung cancer patients
Source: PLoS One. 2021 May 27;16(5):e0252053. doi: 10.1371/journal.pone.0252053 (PMC8158910; doi:10.1371/journal.pone.0252053)
Supplement: S16 Table — (DOCX) [file pone.0252053.s021.docx]

|  | | | | | | | |
| --- | --- | --- | --- | --- | --- | --- | --- |
|  |  | **Cost ($)** | | | | | |
|  |  | *hfIGRT* | *No hfIGRT* | *Difference* | *Lower 95% CI* | *Upper 95% CI* | *P-value* |
| **All Patients** | *IGRT* | $2,248.60 | $197.17 | $2,051.43 | $1,916.46 | $2,179.23 | < 0.01 |
|  | *Radiation* | $19,083.44 | $12,009.1 | $7,074.34 | $6,559.47 | $7,581.49 | < 0.01 |
|  | *All Care* | $74,587.75 | $64,839.15 | $9,748.6 | $6,237.24 | $13,426.86 | < 0.01 |
| **Matched Cohort** | *IGRT* | $1,971.41 | $199.03 | $1,772.38 | $1,605.69 | $1,925.71 | < 0.01 |
|  | *Radiation* | $17,330.31 | $15,024.17 | $2,306.15 | $1,559.12 | $3,046.65 | < 0.01 |
|  | *All Care* | $71,568.63 | $69,693.25 | $1,875.38 | -$3,230.72 | $6,924.93 | 0.49 |
