# Supplementary material for: Efficacy and cost of high-frequency IGRT in elderly stage III non-small-cell lung cancer patients
Source: PLoS One. 2021 May 27;16(5):e0252053. doi: 10.1371/journal.pone.0252053 (PMC8158910; doi:10.1371/journal.pone.0252053)
Supplement: S1 File — (DOCX) [file pone.0252053.s022.docx]

**S1 File. Methods**

**S1A Methods**. Definition of definitive (i.e., non-palliative) radiotherapy.

Prescriptions for definitive radiotherapy vary by institution, physician, and patient-specific circumstances. Common – but certainly not the only – prescriptions for definitive radiation for stage III lung cancer include 6000cGy delivered in 30 once daily fractions to 6660cGy given in 37 once daily fractions. To reduce the likelihood of including patients treated with palliative intent for metastatic disease, we defined a definitive course of radiation as: (i) between 25 and 45 radiotherapy fractions; (ii) completed within 6 months of diagnosis. The number of radiotherapy fractions was calculated by counting the number of unique dates on which clusters of CPT codes for radiation delivery were billed (**S1 Table**). Because patients who recur or develop distant metastatic disease can receive additional radiation, we assumed that (iii) a break of 30 days or more between sequential radiation codes indicated an additional course of radiation. Radiation administered after a 30-day break was not included in the count of total fractions administered.

**S1B Methods**. Motivation and validation of our claims-based definition for hfIGRT.

To create a claims-based definition of hfIGRT, we started by calculating for each patient the ratio of images-to-treatment fractions using the following formula:

$$\% imaging during treatment= \frac{\# images}{\# radiation treatment fractions}$$

An imaging-to-fraction ratio of 100% means a patient received daily imaging with each and every fraction of radiation treatment. A ratio of 0% means a patient never received any imaging with any fraction of radiation treatment.

**S1 Fig** shows the distribution of the ratio of imaging-to-fractions received by patients during radiation treatment. It shows two clear peaks. The first peak, centered around 20%, corresponds to patients that received imaging on one day per week. The second peak, centered around 100%, corresponds to patients that received imaging on each and every weekday. Less than 5% of patients were present in the nadir between the two peaks. We chose a 65% image-to-fraction cutoff for hfIGRT because, by inspection, it approximated the position of this low-point. Hence, a small change in this cutoff in the positive or negative direction would have almost no effect on the number of patients defined as receiving hfIGRT. Furthermore, a 65% cutoff clearly divides the weekly and daily IGRT groups in two.

**S1C Methods**. Description and calculation of covariates.

For each patient, several additional covariates were collected or calculated. We collected patient demographic and tumor information, including age at diagnosis, gender, race, marital status, year of diagnosis, and primary tumor size, grade, and stage from SEER registry data.

Using Medicare claims in the 12 months prior to cancer diagnosis, we calculated the Klabunde adaptation of the Charlson comorbidity index to assess the prevalence of comorbid disease in our cohort [1-4]. We separately used the Centers for Medicaid & Medicare Services (CMS) Chronic Conditions Data Warehouse (CCW) algorithms to identify additional non-Charlson scored comorbid conditions, including chronic obstructive pulmonary disease (COPD) [5].

*Radiation Treatment Characteristics*

Patients were sub-stratified according to whether or not they were treated using intensity-modulated radiation therapy (IMRT). IMRT was defined as the presence of any IMRT planning or treatment billing code during the course of radiation (**S1 Table**).

We separately reported the number of fractions delivered, whether patients received therapy in a hospital or freestanding clinic, and if they received care in a rural or urban setting based on SEER registry data.

*Cancer-related factors and non-radiation treatment*

Using Medicare MEDPAR, OUTSAF, NCH, HHA, and DME claims codes (**S1 Table**), we tabulated diagnosis and staging procedures. In a similar fashion, we recorded treatments given before, after or during radiotherapy, including oxygen, surgery, and/or chemotherapy. We classified patients as treated with intravenous chemotherapy if they received it within 6 months of cancer diagnosis [6].

*Provider volume*

We defined provider volume as the number of patients with NSCLC treated by a physician in a year using the technique developed by Boreo et. al [7]. Existing literature has confirmed a high degree of correlation between patient volumes calculated using this technique and actual physician volume. We identified the specific provider with the older Unique Physician Identification Number (UPIN) or newer National Physician Identifier (NPI) on the weekly management code, 77247. This code has the virtue of being provider specific, unlike other technical codes that link to facilities or organizations. A crosswalk file allowed us to identify patients with both an NPI and a UPIN and prevent us from double counting them. Provider volume was expressed as patients treated per year, which was defined as the total number of patients with NSCLC divided by the time interval between the treatment dates of the first and last patient.

**S1D Methods**. Calculating cost of care

Total costs included Medicare payment aggregated from inpatient facility claims in the Part A Medicare Provider Analysis and Review (MEDPAR) files, outpatient facility claims in the Part B hospital-based Outpatient Claims (OUTSAF) files, and the in- or outpatient physician claims in the Part B Carrier Claims (formerly the Physician/Supplier or NCH) files. Narrower radiotherapy and related costs summed claims based on the CPT codes between 77261 – 77999 in the Outpatient and Carrier Claims files (**S1 Table**).

We adjusted costs for inflation, normalizing them to the year 2017 using the Prospective Pricing Index for Part A claims and the Medicare Economic Index for Part B claims. We simultaneously adjusted costs for geographic variation using the geographic adjustment factor for Part A claims and the Geographic Practice Cost index for Part B claims.

The National Cancer Institute’s Health Services and Economics Branch of the Applied Research Program provided all the adjustments used to tabulate costs.

**S1E Methods**. Using Local Coverage Determinations

A secondary endpoint looked at whether Carrier Local Coverage Determinations (LCDs) correlated with hfIGRT use. Medicare’s administrator, the Centers for Medicare and Medicaid (CMS), uses regional, private companies to handle certain Medicare claims and their processing. Contractors were originally called Fiscal Intermediaries for Part A claims or Carriers for Part B claims. (After passage of the Part D prescription drug amendments in 2003, CMS changed their names to A or B Medicare Administrative Contractors (MACs)). These contractors create LCDs for their coverage area that provides guidance on when it is appropriate to bill under a given CPT code. LCDs cover territories that generally, but not exactly, overlap the 16 SEER registries contained in the SEER-Medicare database.

For our study, we initially looked for – but failed to find any - LCDs governing IGRT use. Hypothesizing that IMRT and IGRT use are correlated, we next sought LCDs covering intensity-modulated radiotherapy (IMRT) billing and binned them into one of three groups as defined by Smith et al.: (1) LCDs that had restrictive IMRT guidance (that expressly permitted only inverse computer planning), (2) LCDs that had permissive IMRT guidance (that allowed both inverse and non-inverse planning), and (3) LCDs that had no IMRT guidance. But why investigate IMRT LCDs in a study of IGRT?

IMRT is a method of radiation delivery that can potentially benefit from more frequent (i.e., high-frequency) IGRT use. In contrast to non-IMRT, IMRT uses more treatment angles and computer-controlled, variable modulation of the treatment beam intensity. On the one hand, these features make IMRT radiation treatment times longer and more expensive. On the other hand, these same features generally make IMRT treatments more precise (or conformal); as a result, oncologists can in theory use much smaller planning treatment volume (PTV) margins. But using smaller PTV margins requires more accurate patient positioning, the very problem IGRT was developed to address. Hence, IGRT use should be closely correlated with IMRT use and this intuition was validated by our logistic regression. Since physicians – not hospitals – ultimately decide whether or not to use IMRT and IGRT, we focused on Carrier LCDs since they govern physician reimbursement (versus hospital reimbursement, which is managed with difference guidelines) and, by extension, shape physician incentives.

Similar to Smith et al, we identified 10 registries (Connecticut, Greater California, Hawaii, Iowa, Los Angeles, New Jersey, New Mexico post-2008, San Francisco, San Jose, Seattle, and Utah post-2007) with restrictive IMRT LCDs during our study period from 2006 – 2013; 3 registries with permissive IMRT LCDs (Detroit, Atlanta, and rural Georgia); and 3 registries (Kentucky, Louisiana, New Mexico pre-2008, and Utah pre-2007) that lacked any IMRT LCDs.

**S1F Methods**. Statistical analysis

*Chi-square bivariate analysis***.** We compared the distribution of patient characteristics between the two treatment groups with the Pearson’s chi-square test.

*Logistic regression testing associations with hfIGRT utilization***.** Bivariate associations at a significance level of p = 0.20 or less were included in an initial multivariable logistic regression model to predict hfIGRT utilization. Logistic models were calculated using both state and LCD as a proxy for geographic location. The model was modified using stepwise forward and backwards elimination with threshold values of p ≤ 0.20 and p ≤ 0.05, respectively. We assessed the quality of our model by checking the area under the curve (c = 0.86 > 0.70 for the state-based model, similar for the LCD model) and the Hosmer and Lemeshow goodness of fit (p = 0.23 > 0.05 or the state-based model, similar for the LCD model).

*Kaplan-Meier analysis testing association between hfIGRT and toxicity***.** The association between hfIGRT and toxicities were assessed using the Kaplan-Meier method with censorship at the earliest of the following: death, or the end of the study period on December 31, 2013. For each endpoint, the proportional hazards assumption with respect to radiation technique was tested visually by inspection of log-log plots and analytically using Schoenfield residuals.

*Cox regression testing associations with toxicity***.** As in our logistic model, Cox regressions were performed using stepwise forward and backwards elimination with threshold values of p ≤ 0.20 and p ≤ 0.05. Clinically important covariates including age, oxygen status, performance status, stage, number of radiation treatments, and treatment strategy were included in the final model regardless of their p-values during selection. For all toxicity endpoints, the proportional hazards assumption with respect to radiation technique was satisfied, and goodness- of-fit for all final models was acceptable (p > 0.05).

*Propensity score matching***.** Based on the results of our logistic regression, we created a propensity score model to validate the findings of the multivariate Cox regressions. Patients were randomly sorted and then matched 1-to-1 without replacement to a nearest neighbor with a match caliper of 0.01. Bivariate association p-values were used to ensure that matched patients were well-balanced across covariates. Proportional hazards models, adjusted for unbalanced covariates (p < 0.20), were generated to compare the cohorts using forward and backward selection as described above. Plots of toxicity on the matched cohorts were re-generated using the Kaplan-Meier method.

*Survival analysis***.** Overall and cause-specific survival was tested in the same way as toxicities with multivariate Cox regressions and matched cohort analysis.

To protect patient anonymity and consistent with policies governing the use of SEER-Medicare data, none of the tables report the number of patients in any sub-group with a sample size less than 11. Analyses were performed with SAS 9.4 (SAS, Cary, NC).

*Estimating the cost differential between the two treatment groups*. A non-parametric bootstrap model used 1,000 samples to estimate the 95% confidence interval (CI) around the mean cost difference between the hfIGRT and non-hfIGRT groups. We estimated these differences for both the original, unmatched cohort and the 1-to-1 matched sub-cohort.

*Wilcoxon bivariate analysis of covariates against cost*. The impact of individual (categorical) covariates on the (continuous) cost of treatment was assessed using the Wilcoxon two-sample test.

To protect patient anonymity and consistent with policies governing the use of SEER-Medicare data, none of the tables report the number of patients in any sub-group with a sample size less than 11. Analyses were performed with SAS 9.4 (SAS, Cary, NC).

**Refrences**

1. <https://healthcaredelivery.cancer.gov/seermedicare/program/comorbidity.html>., N.C.I.D.o.C.C.P.S.S.-M.C.o.C.W.a.

2. Charlson, M.E., et al., *Assessing illness severity: does clinical judgment work?* J Chronic Dis, 1986. **39**(6): p. 439-52.

3. Klabunde, C.N., et al., *A refined comorbidity measurement algorithm for claims-based studies of breast, prostate, colorectal, and lung cancer patients.* Ann Epidemiol, 2007. **17**(8): p. 584-90.

4. Klabunde, C.N., et al., *Development of a comorbidity index using physician claims data.* J Clin Epidemiol, 2000. **53**(12): p. 1258-67.

5. *Centers for Medicare & Medicaid Services (CMS) Chronic Conditions Data Warehouse (CCW) Chronic Condition Algorithms. Definitions at* [*https://www.ccwdata.org/web/guest/condition-categories*](https://www.ccwdata.org/web/guest/condition-categories)*.*

6. Warren, J.L., et al., *Utility of the SEER-Medicare data to identify chemotherapy use.* Med Care, 2002. **40**(8 Suppl): p. IV-55-61.

7. Boero, I.J., et al., *Importance of radiation oncologist experience among patients with head-and-neck cancer treated with intensity-modulated radiation therapy.* Journal of Clinical Oncology, 2016. **34**(7): p. 684-690.
